# Molecular Targeted Intervention for Pancreatic Cancer

**DOI:** 10.3390/cancers7030850

**Published:** 2015-08-10

**Authors:** Altaf Mohammed, Naveena B. Janakiram, Shubham Pant, Chinthalapally V. Rao

**Affiliations:** Center for Cancer Prevention and Drug Development, Department of Medicine, Hem-Onc Section, PC Stephenson Cancer Center, University of Oklahoma Health Sciences Center, Oklahoma City, OK 73104, USA; E-Mails: njanakir@ouhsc.edu (N.B.J.); Shubham-pant@ouhsc.edu (S.P.)

**Keywords:** pancreatic cancer, chemoprevention, chemotherapy, combination treatment, drug development

## Abstract

Pancreatic cancer (PC) remains one of the worst cancers, with almost uniform lethality. PC risk is associated with westernized diet, tobacco, alcohol, obesity, chronic pancreatitis, and family history of pancreatic cancer. New targeted agents and the use of various therapeutic combinations have yet to provide adequate treatments for patients with advanced cancer. To design better preventive and/or treatment strategies against PC, knowledge of PC pathogenesis at the molecular level is vital. With the advent of genetically modified animals, significant advances have been made in understanding the molecular biology and pathogenesis of PC. Currently, several clinical trials and preclinical evaluations are underway to investigate novel agents that target signaling defects in PC. An important consideration in evaluating novel drugs is determining whether an agent can reach the target in concentrations effective to treat the disease. Recently, we have reported evidence for chemoprevention of PC. Here, we provide a comprehensive review of current updates on molecularly targeted interventions, as well as dietary, phytochemical, immunoregulatory, and microenvironment-based approaches for the development of novel therapeutic and preventive regimens. Special attention is given to prevention and treatment in preclinical genetically engineered mouse studies and human clinical studies.

## 1. Introduction

Pancreatic cancer (PC) remains a deadly disease, with uniform and rapid lethality despite six decades of research. PC is the one of the most common cancers diagnosed in the U.S.; the lifetime risk for development of PC in the US is about 1.5%, and it is the fourth leading cause of U.S. cancer deaths [1]. In 2015, it is expected that 48,960 patients will be diagnosed with PC and 40,560 will die, most within the first year of diagnosis. The five-year survival rate for PC patients is less than 6%, with a median survival of 4–6 months. Over the past decade, PC death rates have increased, whereas a downward trend has been observed for most other cancers, such as colorectal, breast, lung, and prostate cancers. Patient survival is marginally improved with the FDA-approved drug gemcitabine and/or with other cancer drugs, such as oxaliplatin, cisplatin, or erlotinib [2,3,4,5,6]. Despite aggressive treatment strategies, the use of advanced surgical techniques and post-operative care, and adjuvant therapies, all patients who undergo resection will also eventually succumb to recurrent and/or metastatic disease.

While the life expectancy of patients with pancreatic cancer is certainly low, R0 surgery at an early stage remains the only curative option for treatment. The exact causes of PC are poorly understood, though multiple factors are known to increase risk. PC risk is associated with westernized diet, tobacco, alcohol, obesity, chronic pancreatitis, and family history of pancreatic cancer. Smoking and alcohol use promote PC at an early age; the combination of smoking and alcohol synergistically enhances PC promotion. Smokers have a higher risk of developing PC than non-smokers. One in four or five cases of pancreatic cancer is estimated to be caused by tobacco smoking. Smoking is also associated with early age at diagnosis. Thus, cigarette smoking is the leading preventable cause of pancreatic cancer. The high mortality rate is due to difficulties in establishing an early and accurate diagnosis, since PC is asymptomatic until the advanced stages, in addition to a lack of effective treatment strategies [7,8]. Early detection of pancreatic ductal adenocarcinoma (PDAC) would dramatically improve the overall survival rate [9]. Molecular biomarkers that can improve current PC detection would have great value in improving the survival of patients with PDAC. Early detection of PC, which is expected to result in less extensive treatment and better outcomes, can also be achieved through research on the pathobiology of PC, the identification of early detection strategies, the development of ideal mouse models that resemble human disease progression for effective screening of drugs, and the identification of potential novel targets and drugs.

The unusual resistance of this disease to potential chemotherapeutic drugs is a major challenge. Although *in vitro* assays and *in vivo* cell transplantation models for PC have been extensively employed, these models do not represent the full complexity of the tissue organization, physical barriers, and potential targets that might be exploited for clinical benefit. Transplanted PC cells readily respond to conventional therapeutic agents, in marked contrast to autochthonous tumors in mice and humans. However, this problem was overcome with recent genetically engineered mouse (GEM) models that were developed to understand PDAC development and its pathobiology, and that recapitulate human disease progression. These GEMs are used to study the molecular biology, experimental therapeutics, prevention, and genetic susceptibility and risk. These models have been shown to be useful in the validation of gene function and the identification and characterization of new genes and biomarkers. Research using GEM models has provided insight into the molecular and cellular mechanisms underlying the initiation of pancreatic precursor lesions and the multistage processes leading to the development of PDAC. Better models allow for the testing of novel prevention and therapeutic strategies. This review provides updated information on chemopreventive and therapeutic strategies and clinical and preclinical drug development for PC.

## 2. Molecular Pathobiology of Pancreatic Cancer and Genetically Engineered Mouse Models

Mutations in the Kirsten Rous sarcoma (Kras) virus oncogene are observed in more than 95% of patients with PC. The molecular pathobiology of PC is complex, with different molecular events occurring at various stages of disease progression. PDAC is the most common pancreatic exocrine tumor, representing about 95% of cases. The details of the genetic alterations and mechanisms of initiation and development of PDAC have been extensively studied [9,10,11]. Initiation of the disease begins with a mutation, usually at codon 12, in the Kras gene in the normal pancreatic cell.

There is some controversy regarding whether PC arises from acinar or ductal cells. Recent lineage studies suggest that PC may originate from the acinar cell-associated Kras mutation. Upon Kras mutation, pancreatic acinar cells will transform to duct-like cells in a process called acinar-to-ductal metaplasia (ADM). These duct-like cells will progress into metaplastic ductal lesions or pancreatic intraepithelial neoplasia (PanIN). The morphological and molecular transformation of the mutated cell forms pancreatic precursor lesions: pancreatic intraepithelial neoplasia (PanIN), mucinous cystic neoplasms (MCN), and intraductal papillary mucinous neoplasms (IPMN) that progress to invasive carcinoma [12]. PanIN lesions arise in the small ducts of the pancreas and are the most common precursor lesions. PanIN-1A lesions are characterized by a transition from a normal phenotype with abundant supranuclear, mucin-containing cytoplasm to basally located nuclei and slight nuclear atypia. The development of these lesions is restricted to the small, intralobular ducts of the pancreas in the transgenic mice, just as occurs in the human condition. PanIN1B lesions are identified by the development of papillary or micropapillary ductal lesions without significant loss of polarity or nuclear atypia. As the mice age, higher-grade PanINs will be observed with increasing frequency. In PanIN-2 lesions, moderate nuclear atypia and a loss of polarity occurs. In PanIN-3 lesions, significant nuclear atypia and complete loss of polarity are observed, such that it is often impossible to discern luminal from basal boundaries within the center of one of these lesions. The goblet mucus-producing cells are seen in PanINs of various stages, but most frequently in PanIN-3 lesions, and are considered hallmarks of human PanINs. In PanIN-3 lesions, nuclear enlargement and pleomorphism are apparent; in addition, there are clusters of cells budded off into the lumen, representing cardinal features of human PanIN-3 lesions, also known as carcinoma *in situ*. At later stages, the pancreata contain extensive ductal lesions, and the acinar parenchyma will be largely replaced by an intense stromal, or desmoplastic, reaction comprised of inflammatory cells, fibroblasts, and collagen deposition. This fibroinflammatory reaction is highly reminiscent of that seen in human PC and may be secondary to luminal obstruction from the intraductal proliferations.

These PanIN lesions show varied molecular alterations as they progress to PDAC. Several specific pathways are often altered; these pathways offer potential therapeutic and/or chemopreventive targets. Molecular aberrations occurring during the stepwise development of ductal adenocarcinoma have been studied extensively using preclinical GEM models [13,14,15,16,17,18,19]. After the initial oncogenic mutations, predominantly in the Kras oncogene, PanINs develop. As the disease progresses to PDAC, each PanIN stage is well characterized by multiple molecular alterations with significant genetic irregularities affecting signaling pathways. These irregularities lead to instability of several molecular processes, then to aberrations in the cell cycle, cell growth, and division, leading to aggressive malignant tumor development. At this point, the PDAC cells will achieve self-sufficiency in growth signals, insensitivity to antigrowth signals, evasion of apoptosis, limitless replicative potential, induction of angiogenesis, and capacity for invasion and metastasis. The mutations occurring in PDAC lead to huge genetic heterogeneity of PDAC. A set of 12 core-signaling pathways have been reported to be altered in 69%-to-100% of all PDACs analyzed [20]. These signaling pathways are: apoptosis pathway, DNA damage control pathways, regulation of G1/S phase transition, Hedgehog, hemophilic cell adhesion, integrin, c-Jun N-terminase kinase, Kras, regulation of invasion, small GTPase-dependent, TGF-β, and Wnt/Notch [20]. Molecular pathobiology studies provided significant information on PC precursor lesions implicated with molecular changes at genetic, transcriptomic, epigenetic, and proteomic levels that correlate with histological stages of PDAC development [13,14,15,16,17,18,19]. Several of these pathways have been studied extensively and are targeted in the development of therapeutic and chemoprevention strategies. Extensive studies on molecular signaling in PC have led to some progress in the diagnosis, staging and treatment of localized PC. However, detection of PC at an early stage is not possible using the currently available modalities. The carbohydrate antigen 19-9 (CA19-9), phage display approach used for screening and detection of genetic markers such as Kras and p53 in stool samples are very few biomarkers for PC [21,22,23,24,25,26,27,28].

The identification of several morphological and molecular alterations in pancreatic carcinogenesis has led to development of several complex GEM models of PC that recapitulate key aspects of human PC development, including the development of precursor lesions. All of the developed GEMs show: (a) expression of an oncogenic Kras (Kras^G12D^) in the pancreata of developing mice whose Kras levels are regulated by endogenous promoter elements, rather than by transgenic overexpression, and (b) targeting the oncogenic Kras to a putative progenitor cell population in the pancreas by expressing Cre recombinase under Pdx1 or p48 regulatory elements. Pdx1 and p48 are transcription factors expressed in the developing pancreas that define both endocrine and exocrine progenitor cell populations. Misexpression of a mutant Kras in these cells results in the generation of PanINs and invasive ductal adenocarcinomas. While expression of mutant Kras is by itself sufficient to generate the entire histological spectrum of PanIN lesions, the penetrance of developing invasive cancer, the time to progression to invasive cancer, and the predominant histology of the cancers that arise vary depending on additional genetic alterations, such as mutation of Trp53 or loss of Cdkn2A/Ink4 function, that are engineered simultaneously with Kras. These GEM mice also display many molecular aberrations in developmental signaling pathways, such as Notch and Hedgehog. New models like Ela-Kras, Mist1-Kras, and LSL-Kras^G12D/p^; DPC4^flox/p^ that describe and mimic human IPMN and MCN have also been engineered [29,30,31,32,33].

Not only do the PanIN lesions in the various LSL-Kras^G12D^ mice demonstrate the morphological spectrum of human PanIN lesions, they also carry many of the alterations described above, such as overexpression of Notch and Hedgehog gene targets, cyclooxygenase-2 (COX-2), and matrix metalloproteinase-7 (MMP-7) [34]. The use of these mice has helped to establish PanINs as the precursors to invasive adenocarcinoma of the pancreas, and also provides an unprecedented opportunity to study the basic biology and translational aspects of PC and its precursor lesions in a controlled *in vivo* setting [34]. Understanding the developmental biology leads to identification of important markers in the PanIN lesions that can be used for development of better prevention and treatment strategies and of modalities that can detect PanIN biomarkers. The development of GEM models of PDAC has significantly advanced our ability to delineate important molecular and pathological changes that drive pancreatic carcinogenesis. Identification of these aberrant molecules will help to address issues contributing to the lethality of this disease and will provide targets for effective intervention and treatment. The successful evaluation of novel drugs for chemoprevention and treatment of PC has been made possible by the development of several GEM models.

## 3. Treatment Strategies: Chemotherapy and Chemoprevention

Recent studies demonstrate that pre-invasive PanINs progress slowly to invasive PC [35]. This slow progression is underscored by recent data showing that PanIN progression, from the initiating mutations through the emergence of the parental founder cells of invasive carcinoma within the high-grade PanIN lesion to acquisition of metastatic capacity, takes several years [35]. Thus, there is a several-year time frame for effective intervention with prevention and treatment strategies. Despite advances in the field of molecular genetics in human PC, targeted therapies have not yet translated into improved overall survival (OS) for patients. Current treatment modalities for advanced PC include gemcitabine as a single agent or in combination with erlotinib or nab-paclitaxel, or a combination of 5-fluororuacil (5FU), leucovorin, oxaliplatin, and irinotecan (FOLFIRINOX). In spite of recent advances, the median OS remains modest, varying from 5.6 to 11.1 months [2,36,37,38].

**Figure 1:**
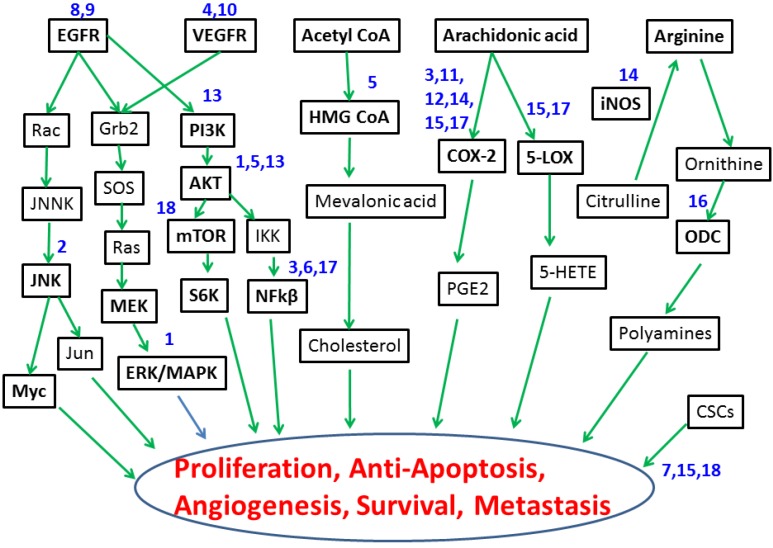
Molecular targets for pancreatic cancer prevention and therapy. Drugs evaluated using transgenic mouse models include: (**1**) Vitamin E/δ-tocotrienol, (**2**) JNK inhibitor, (**3**) DMAPT (dimethylaminoparthenolide) + Sulindac, (**4**) B 20-4.1.1, (**5**) Atorvastatin, (**6**) Capsiacin, (**7**) Resveratrol, (**8**) Gefitinib, (**9**) Erlotinib, (**10**) Bevacizumab, (**11**) Celecoxib, (**12**) Nemesulide, (**13**) GDC 0941, (**14**) NO-Aspirin, (**15**) Licofelone, (**16**) DFMO, (**17**) Curcumin, and (**18**) Metformin.

Patients with PDAC often present with distant metastatic disease. Among patients with metastatic PDAC, there has been a modest and disappointing improvement in OS over the past two decades. More effective therapeutic strategies are desperately needed. Hence, developing strategies and identifying novel therapies that delay/inhibit/prevent progression to PC and those that can treat existing PC by targeting defective signaling molecules and/or tumor microenvironment is of the utmost importance (Figure 1).

Mounting evidence suggests that targeting genetic abnormalities improves the treatment response in several human cancers. Many of the genetic alterations occu during multi-step progression of pre-malignant precursor lesions into invasive adenocarcinoma. The advent of GEMs has revealed molecular mechanisms and potential targets underlying the interactions among various genetic alterations in pancreatic tumorigenesis. Potential molecularly targeted treatment strategies that are effective for PC prevention and therapy (Figure 1) in preclinical transgenic mouse models and clinical settings are presented in Table 1 below.

**Table 1:** GEM models for evaluation of drugs for pancreatic cancer prevention and treatment.

	GEMs	Drug	Target	Comment
1	LSL-Kras^G12D/+^; Pdx-1^Cre^; Kras^Cre^ (KC); Kras-p53^Cre^ (KPC)	Vitamin E δ-tocotrienol	MEK/ERKPI3K/AKT	Prolonged survival and delayed PanIN progression
2	Ptf1a; LSL-Kras; Tgfbr2	JNK inhibitor	JNK	Decreased pancreatic cancer and prolonged survival
3	KRas^G12D^; Trp53^R172H^; Pdx-1^Cre^	Minnelide	-	Reduced pancreatic tumor growth and spread, improved survival
4	LSL-Kras^G12D^; Pdx-1^Cre^	DMAPT + Sulindac, DMAPT + Gemcitabine, Sulindac + Gemcitabine, DMAT + Sulindac + Gemcitabine	COX-2, NFkβ	Delayed/prevented progression of PanIN lesions
5	eNOS^−/−^ KC (LSL-Kras^G12D/+^; Pdx-1-Cre^tg/+^)	l-NAME	eNOS	Decreased PanINs and PDAC
6	LSL-Kras^−G12D^; p16/p19^fl/fl^; Pdx1^Cre^	B20-4.1.1, B20-4.1.1+ Gemcitabine	VEGF	No effect on metastasis Significant overall survival (Prevention setting)
7	LSL-Kras^G12D^; LSL-Trp53^R172H^-Pdx1^Cre^	Atorvastatin	HMG-COA	Increased survival and decreased tumor volume
8	p48^Cre/+^-LSL-Kras^G12D/+^	Atorvastatin	PI3K/AKT	Delayed PanIN progression to PDAC, decreased tumor weight
9	LSL-Kras^G12D^/Pdx1^Cre^	Capsiacin	NFKβ, AP-1	Decreased PanIN lesions
10	Pdx1^Cre^; LSL-Kras^G12D^	Resveratrol	CSCs	Decreased size and weight of pancreas
11	LSL-Kras^G12D/+^	Gefitinib	EGFR	Prevented PanIN progression to PDAC, reduced tumor growth
12	LSL-Kras^G12D/+^; LSL-Trp53^R127H/+^; Pdx-1^Cre^ (KPC)	CCDO-Me, LG268, CCDO-Me + LG268, CCDO-ehtyl amide + LG268	-	Prolonged survival by 3–4 weeks
13	Kras^LSL−G12D^; p16/p19^fl/fl^; Pdx1^Cre^	Gemcitabine, Erlotinib, Bevacizumab	EGFR, VEGF	Increased survival advantage
14	Pdx1^Cre^; Z/EGFP; LSL-Kras^G12D/+^; LSL-Trp53^R172H/+^	Dasatinib	Src	Inhibited development of metastasis
15	LsL-Kras^G12D^; Pdx1^Cre^; LSL-Kras^G12D^; LSL-Trp53^R172H^; Pdx1^Cre^	Enapril Aspirin Enapril + Aspirin	ACE	Delayed progression of PanIN lesions
16	EL-Kras	Menhaden Oil	-	Decreased precancerous lesions
17	Kras p53^L/+^	GS1	Gamma secretase	Inhibited tumor development
18	PDA.Muc1	Muc1 vaccine, celecoxib, Muc1 vaccine + celecoxib, Muc1 vaccine + celecoxib + Gemcitabine	T-reg, MSC, COX-2	Decreased tumor weights, PanIN lesions, and invasive carcinoma
19	Pdx1-Cre; LSL-Kras^G12D^; Ink4a/Arf(lox/lox)	Cyclopamine	Hedgehog	Prolonged median survival
20	KRas ^G12D^; Pdx1^Cre^	Nemesulide	COX-2	Delayed progression of PanIN lesions
21	Ptf1a^cre/+^; LSL-Kras^G12D^; Trp53^R172H/f^	GDC 0941	PI3K/AKT	Blocked tumor growth
22	Fat-1-p48^Cre/+^-LSL-Kras^G12D/+^	Endogenous n-3 fatty acids		Delayed PanIN progression to PDAC and decreased tumor weights
23	p48^Cre/+^-LSL-Kras^G12D/+^	NO-Aspirin	COX-2, iNOS	Inhibited PanIN 3 lesions and PDAC
24	p48^Cre/+^-LSL-Kras^G12D/+^	Licofelone	COX/5-LOX	Prevents PanIN and PDAC, decreased tumor weights
25	p48^Cre/+^-LSL-Kras^G12D/+^	Licofelone + Gefitinib	COX/5-LOX, EGFR	Reduced PanIN lesions, PDAC incidence, carcinoma spread
26	p48^Cre/+^-LSL-Kras^G12D/+^	DFMO	ODC	Reduced PanIN lesions, PDAC incidence, carcinoma spread
27	p48^Cre/+^-LSL-Kras^G12D/+^	Curcumin	COX-2, 5-LOX, NFkβ	Reduced PanIN lesions, PDAC incidence (unpublished)
28	p48^Cre/+^-LSL-Kras^G12D/+^	Metformin	mTOR, AMPK, CSCs	Reduced PanIN lesions, PDAC incidence

## 4. Molecularly Targeted Treatment Strategies

### 4.1. Kras-Targeted Treatment

The first molecular aberration observed during transformation of a normal cell into a PC precursor is Kras mutation. More than 95% of PCs are associated with Kras oncogenic mutations. The Kras gene encodes a RAS protein that belongs to the guanosine triphosphatase (GTPase) family. A single point mutation at codon 12 is most common in PC; it results in one amino acid substitution that causes constitutive activation of Kras [7,8,39]. This mutation leads to upregulation/dysregulation of the epidermal growth factor receptor (EGFR) and induction of proliferation by activation of mitogenic pathways. No effective inhibitor of Kras has yet been developed for translation into clinical studies. However, therapeutic strategies have focused on developing inhibitors that interfere with farnesylation of Kras, which is required for its activation. Tipifarnib (R115777), a farnesyltrasferase (FTase) inhibitor, prevents activation of RAS and downstream signaling. Tipifarnib, alone or in combination with gemcitabine, did not provide any additional benefit to patients with advanced PC [40,41,42]. However, combinations of FTase and geranylgeranyl transferase (GGTase) dual inhibitors showed minimal efficacy when used along with radiation therapy for patients with locally advanced pancreatic adenocarcinoma [43]. Peptide vaccines against mutant Kras have been shown to invoke immune responses directed at PC. In the adjuvant setting, combinations of mutant Kras peptide with granulocyte-macrophage colony-stimulating factor (GMCSF) were intradermally administered as a vaccine to patients with resected pancreatic tumors. Fifty-eight percent of patients showed an induction of peptide-specific immunity, with a significant increase in median survival [44]. The combination of mutated Kras vaccine with gemcitabine led to a 2.4-month increased median survival in patients with R1 resection status PC [45]. Administration of mutated Kras vaccine combined with gemcitabine thus merits consideration for inclusion in treatment protocols for PC. Immunological studies performed during this clinical trial revealed that the patients who received the vaccine had significantly higher T-cell responses specific to the Kras vaccine antigens compared with those receiving placebo.

### 4.2. EGFR-Targeted Treatment

Activation of EGFR, a cell surface receptor tyrosine protein kinase in the Erb B family, controls proliferation and differentiation of epidermal and mesenchymal cells. Several clinical studies and preclinical GEM (genetically engineered mouse) studies have shown overexpression of EGFR in PC. Constitutive activation or amplification of EGFR is also a common PC feature. EGFR has been exploited as a therapeutic target for monoclonal antibodies. Genetic ablation or pharmacological inhibition of EGFR effectively reduces Kras-driven tumorigenesis. Using GEMs and pharmacologic inhibition of EGFR, Navas *et al.* and Adrito *et al.* recently showed that EGFR signaling is essential for Kras-driven pancreatic tumorigenesis [46,47]. Several EGFR inhibitors have been tested at preclinical and clinical levels. Monoclonal antibodies, like cetuximab and panitumumab, prevent EGFR-induced receptor activation and dimerization. However, the combination of cetuximab with cytotoxic drugs did not provide additional survival benefits to patients with advanced carcinoma [48,49,50]. Erlotinib and gefitinib are oral small molecules that inhibit the tyrosine kinase activity of EGFR. A statistically significant survival advantage was observed in patients treated with a combination of erlotinib and gemcitabine over gemcitabine treatment alone [36]. Preclinical studies in the p48^Cre/+^-LSL-Kras^G12D/+^ mouse model showed a significant decrease in PDAC progression upon gefitinib treatment in the early stages of cancer development [51]. Hence, EGFR can serve as a valuable target for PC chemoprevention and chemotherapy.

### 4.3. COX-2-Targeted Treatment

Cyclooxygenase (COX) enzymes often are up-regulated in cancer. COX-2 catalyzes formation of prostaglandins that play a role in inflammation, cancer progression, and chemoresistance. COX-2 up-regulation has been reported in 56%-to-90% of pancreatic adenocarcinomas, and in 65% of PanINs [52,53,54,55,56,57,58]. However, a phase II clinical trial using a combination of celecoxib, gemcitabine, and cisplatin in patients with metastatic PC suggested that celecoxib did not provide additional benefits [59]. A phase II clinical study of celecoxib and gemcitabine showed that patients with advanced PC had a median survival of 9.1 months and an overall clinical response of 54%, indicating some clinical benefits [60]. The efficacy and toxicity of a short intensive Uracil/Tegafur (UFT)-based chemoradiotherapy scheme combined with celecoxib was investigated in locally advanced PC. Full treatment compliance was achieved in 55% of the patients; 80% received at least three weeks of treatment. Median survival was 10.6 months; median time to progression was 6.9 months. The substantial toxicity of mainly gastrointestinal origin, the lack of response, and the reported mediocre OS and PFS does not warrant pursuance of this treatment strategy [61].

In preclinical GEM models, a combination of a COX-2 inhibitor (celecoxib) and low-dose chemotherapy (gemcitabine) with a novel Mucin-1 (MUC1)-based vaccine was effective in preventing the progression of PanIN lesions to invasive PDAC in an appropriate human MUC1-expressing transgenic mouse model of PC induced by Kras (G12D) mutation. The combination treatment elicited robust antitumor humoral and cellular immune responses with increased tumor apoptosis. Mechanistically, the increased immune response was due to the downregulation of indoleamine 2,3-dioxygenase enzymatic activity and circulating prostaglandin E_2_, and reduced levels of myeloid suppressor and T regulatory cells in the tumor microenvironment [62]. Intervention with dimethylaminoparthenolide (DMAPT) and sulindac combined with gemcitabine reduced progression of premalignant pancreatic lesions in the LSL-Kras^G12D^; Pdx-1^Cre^ mouse model of PC [63]. Funahashi *et al.* found that the selective COX-2 inhibitor nimesulide delays the progression of PC precursor lesions in a conditional Kras^G12D^ mouse model [64]. Rao *et al.* used GEM p48^Cre/+^-LSL-Kras^G12D/+^ mice to evaluate the potential chemopreventive effects of the nitric oxide-releasing NSAID, NO-Aspirin, against pancreatic tumor progression, and observed a significant decrease in tumor progression and reduced cyclooxygenase-2 expression and activity [65].

### 4.4. 5-Lipoxygenase-Targeted Treatment

Increasing evidence implicates 5-lipoxygenase (5-LOX) in the growth of several tumor types, including pancreatic, colorectal, prostate, and breast cancer. Numerous studies have demonstrated overexpression of 5-LOX in tissue samples of primary tumor cells and in established cancer cell lines [66]. Like COX-2, 5-LOX and its metabolites, 5-hydroxyeicosatetraenoic acid (5-HETE) and leukotriene B_4_ (LTB_4_), play important roles in PC [67,68,69]. 5-LOX overexpression is well documented in human pancreatic lesions, patients with chronic pancreatitis, GEM lesions, and chemical-induced PC in hamsters [70]. We have recently shown that the dual COX-5-LOX inhibitor licofelone will prevent progression of PanIN lesions to PDAC in genetically engineered Kras^G12D/+^ mice [71]. No clinical studies have yet been undertaken using 5-LOX inhibitors for PC prevention and treatment.

### 4.5. PI3K/AKT/mTOR Signaling-Targeted Treatment

One of the three important mitogenic signaling pathways that EGFR uses to promote cell division involves activation of phosphotidylinositol-3 kinase (PI3K). Significant evidence points to PI3K as a promising signaling node for targeted therapeutic intervention. Markers that may predict responsiveness of PC cells to PI3K inhibitors have not yet been identified, but are needed for better stratification of patients in clinical trials. PI3K activation leads to activation of protein kinase B (AKT), a serine/threonine kinase that leads to activation of mammalian Target of Rapamycin (mTOR), another serine/threonine protein kinase. The PI3K/Akt/mTOR signaling pathway is involved in various solid tumors, including advanced PC. In PC, Kras mutation leads to aberrant activation of cell proliferation pathways, including PI3K/Akt/mTOR signaling. More than half of all patients with PC have activation of the PI3K/Akt/mTOR pathway, which correlates with a poorer outcome. The AKT inhibitor nelfinavir, in combination with gemcitabine, cisplatin, and radiotherapy, has been used in Phase I clinical trials in patients with locally advanced PC. Complete regression was observed in more than 50% of the patients who completed chemoradiotherapy [72]. The mTOR inhibitor temsirolimus, an analog of rapamycin, did not produce any response or stability of the disease in a Phase II clinical trial [73]. The mTOR inhibitor everolimus, which has immunosuppressive and anti-angiogenic activities, binds to the rapamycin (FK506) binding protein (FKBP)-12. The resulting complex binds to and inhibits mTOR. However, the combination of everolimus with erlotinib did not produce any response or disease stability in a Phase II clinical trial of advanced pancreatic carcinoma [74].

The chemopreventive efficacy of atorvastatin against the progression of PanINs to PDAC and its effect on the PI3K/AKT signaling pathway were evaluated in conditional p48^Cre/+^-LSL-Kras^G12D/+^ transgenic mice [75]. Mice fed 200 and 400 ppm atorvastatin had a PDAC incidence of 65% and 35%, respectively, compared with 85% incidence in untreated controls. A significant suppression of PanIN-3 (22.6%) was observed in mice fed high-dose atorvastatin, with ~68% of the pancreata free from ductal lesions. Pancreata of mice administered atorvastatin had significantly reduced expression of phospho (p)-extracellular regulated kinase (ERK), AKT, and p-AKT. This study showed that atorvastatin significantly delays the progression of PanIN-1 and -2 lesions to PanIN-3 and PDAC coincident with modulation of PI3K/AKT signaling molecules in a preclinical model, suggesting potential clinical benefits of statins for patients with high-risk PC [75]. Atorvastatin also inhibited carcinogenesis and increased survival in LSL-Kras^G12D^ Trp53^R172H^-Pdx1^Cre^ mice [76]. Similarly, the antidiabetic drug metformin was found to delay significantly pancreatic lesion progression to PDAC by inhibiting mTOR and increasing phospho-adenosine monophosphate kinase (pAMPK) [77]. These studies underscore the importance of targeted strategies for PC prevention (Table 1, Figure 1).

### 4.6. Combination Treatment

Recently, several clinical trials have been published on gemcitabine-based chemotherapy, with or without the addition of different agents, in patients with advanced PC, with diverse results. Meta-analysis indicates that gemcitabine as adjuvant therapy after the resection of PC prolongs OS, compared with other treatments [78]. Recently, an open-label, randomized, multi-center phase II trial explored the role of maintenance sunitinib after first-line chemotherapy. The primary end-point was fulfilled, and the 2-year OS was remarkably high, suggesting that maintenance sunitinib is promising and should be further explored in this patient population [79]. A combined chemoprevention approach using several agents that affect multiple targets is an emerging strategy to combat PC at early stages [80,81]. Some agents being tested in clinical studies target more than one molecule. These agents may have enhanced efficacy to control the growth of malignant PC.

#### 4.6.1. Preclinical Updates on Combination Treatment Strategies:

Several studies in preclinical GEM models support the use of combinations of agents to target multiple genetic defects and combat PC progression. Dasatinib significantly inhibited the development of metastases in Pdx1^Cre^, Z/EGFP, LSL-Kras^G12D^, and Trp53^R172H^ mice [82]. Treatment with aspirin and enalapril prolonged the median survival of LSL-Kras^G12D^-LSL-Trp53^R172H^-Pdx1^Cre^ transgenic mice compared with untreated mice [83]. Some non-cytotoxic drugs, the synthetic oleanane triterpenoids (CDDO-Me and CDD-ethylamide) and the rexinoid LG100268, were evaluated individually and in combination for the prevention of pancreatic carcinogenesis in the highly relevant LSL-LSL-Kras^G12D^-LSL-Trp53^R172H^-Pdx1^Cre^ (KPC) mouse model of PC. CDDO-Me, LG268, the combination of CDDO-Me and LG268, and the combination of CDDO-ethylamide and LG268 significantly (*p* < 0.05) increased survival in the KPC mice by 3 to 4 weeks (Table 1) [84]. The chemopreventive activity of δ-tocotrienol, a bioactive vitamin E derivative extracted from palm fruit, was evaluated in the LSL-Kras^G12D^-Pdx1^Cre^ PC mouse model. Mice treated with δ-tocotrienol showed increased median survival, with a significant decrease in invasive cancer, and suppression of mouse (m)PanIN progression with inhibition of mutant Kras-driven pathways, such as mitogen-activated kinase effector kinase (MEK)/ERK, PI3K/AKT, and nuclear factor (NF)-kB/p65, as well as decreased Bcl-xL and induction of p27 [85]. The combination of gemcitabine and δ-tocotrienol prolonged survival in KPC transgenic metastatic PC mice, leading to a reversal of epithelial-to-mesenchymal transition (EMT) in tumors [86]. δ-tocotrienol led to increased survival of the transgenic mice and potentiated the antitumor activity of gemcitabine (Table 1). This compound was also observed to reduce angiogenesis, inhibit tumor proliferation, reverse EMT, disrupt oncogenic Kras signaling, and induce apoptosis in mouse pancreatic tumors [86].

We have studied the chemopreventive effects of the combination of licofelone and gefitinib on PanIN lesion progression to PDAC in p48^Cre/+^-LSL-Kras^G12D/+^ mice. Compared with the individual agents, lower doses of the combination caused a significant reduction in PC development. Erlotinib has also been shown to prolong survival in PC by blocking gemcitabine-induced mitogen-activated protein kinase (MAPK/ERK) signals [87]. Of all of the regimens used, the erlotinib and gemcitabine combination provided therapy superior to gemcitabine alone, in both humans and GEMs.

Similarly, capecitabine (CAP), a 5-FU pro-drug approved for the treatment of several cancers, has been used in combination with gemcitabine to treat patients with PC. In both LSL-Kras^G12D^; LSL-Kras^G12D^-LSL-Trp53^R172H^-Pdx1^Cre^ (KPC) mice and subcutaneous allografts of a KPC PDAC-derived cell line (K8484), CAP alone provided 5-FU concentrations in the tumors of ~25 µM, and induced survival similar to gemcitabine in KPC mice, suggesting similar efficacy [88]. The use of multiple agents with several targets may be an ideal strategy for effective prevention and treatment of PC without undue side effects [89].

#### 4.6.2. Clinical Updates on Combination Treatment Strategies

Clinical studies involving sunitinib, which targets the platelet-derived growth factor receptor (PDGFR), vascular endothelial growth factor receptor (VEGFR), KIT, RET, and FLT3; axitinib, which targets VEGFR 1,2,3; and enzastaurin, which targets PI3/AKT and protein kinase Cβ (PKCβ), all failed to increase the survival of patients with PC. The use of axitinib with gemcitabine showed a marginal, but non-significant, survival benefit [90,91,92]. New strategies to prolong disease control warrant investigation in patients with metastatic pancreatic adenocarcinoma. Several other small molecule multi-targeting inhibitors that currently are undergoing clinical trials include: (1) dasatinib and saracatinib, which inhibit SRC and ABL, (2) sorafinib, which inhibits RAF, VEGFR, and mTOR; brivanib, which inhibits fibroblast growth factor receptor (FGFR-1 and -2) and VEGFR-2, (3) vandetinib, which inhibits VEGFR-2, EGFR, and RET, (4) imatinib, which inhibits ABL, PDGFR and KIT, and (5) vatalanib, which inhibits KIT, VEGFR, and PDGFRβ. A retrospective study investigated the impact of angiotensin I-converting enzyme inhibitors (ACEIs) and angiotensin II type-1 receptor blockers (ARBs) in patients with PC who were receiving gemcitabine monotherapy. The ACEIs/ARBs combined with gemcitabine significantly improved clinical outcomes and survival in patients with advanced PC [93].

Recently, several clinical trials used a combination of drugs, with or without gemcitabine, for the treatment of patients with locally advanced and/or metastatic PC. S-1 (TS-1; Taiho) is a newly discovered oral fluoropyrimidine that inhibits dihydropyrimidine dehydrogenase and consists of tegafur, 5-chloro-2,4-dihydroxypyridine, and potassium oxonate at a molar ratio of 1:0.4:1. A phase II trial of gemcitabine and S-1 was conducted to evaluate efficacy and safety as first-line chemotherapy for patients with locally advanced or metastatic PC. None of the patients showed complete response, but some achieved partial response with the combination regimen [94]. The median PFS of patients with advanced PC who were ineligible for a clinical trial was higher in those receiving combination therapy with gemcitabine plus S-1 than in those receiving either drug alone. In the clinically eligible arm, median PFS was 4.5 months, and median OS was 10.5 months. In the clinically ineligible arm, median PFS was 1.1 months, and median OS was 2.9 months. The outcomes for patients who did not meet the eligibility criteria were poor [95]. Hence, it is important to determine which patients could benefit from chemotherapy *vs.* optimal supportive care.

To test whether regional chemotherapy will overcome PC chemoresistance, a prospective phase II study was conducted with a combination of intra-arterial (i.a.) and systemic chemotherapy with 37 treatment cycles in 17 patients. Treatment consisted of an i.a. infusion of 8.5 mg/m^2^ mitomycin C (MMC) and 500 mg/m^2^ gemcitabine through an angiographic catheter into the celiac artery on days 1 and 22, and intravenous infusions of 500 mg/m^2^ gemcitabine on days 8 and 15. The median actual PFS and OS were 4.6 and 9.1 months, respectively. Patients without distant metastases had a longer median survival (15 months) than those with distant metastases (7.1 months) [96]. This combination treatment was well tolerated, and resulted in tumor response rates and median OS and PFS rates that were superior to systemic gemcitabine chemotherapy, and comparable to the more toxic FOLFIRINOX regimen. First-line treatment with FOLFIRI produced a response rate of 25% in patients with PC. In patients receiving FOLFIRI as second-line treatment, 42.9% obtained stable disease as a best response. In the first-line and the second-line cohorts, the median PFS rates were 3.1 months and 3.5 months, respectively, and the median OS rates were 14.5 months and 6.2 months, respectively [97]. FOLFIRI was well tolerated, with good activity as a first-line treatment.

Since 80% of patients with resected PDAC experience treatment failure within two years, the use of preoperative fixed-dose rate gemcitabine combined with the angiogenesis inhibitor bevacizumab and accelerated 30 Gy radiotherapy was studied in an attempt to improve outcomes among patients with potentially resectable PC. Van Buren *et al.* conducted a phase II trial with gemcitabine plus bevacizumab followed by accelerated radiotherapy. Induction therapy with fixed-dose rate gemcitabine and bevacizumab, followed by accelerated bevacizumab/radiotherapy to 30 Gy, was well tolerated [98]. Although effectiveness criteria were achieved, survival outcomes were equivalent to published regimens, indicating no further additional benefit. The efficacy and toxicity of single-agent gemcitabine *vs.* gemcitabine plus cisplatin (G + C) was compared in patients with metastatic PC. The median survival and median time to progression were 7.7 and 4.6 months for gemcitabine alone, and 7.9 and 3.6 months for G + C, respectively. Clinical benefit was 36% for gemcitabine alone and 29% for G + C. Gemcitabine alone and G + C had comparable and modest response rates in metastatic PC, but gemcitabine alone produced fewer toxicities than did G + C, indicating that caution must accompany the use of these combination treatments [99]. In a single-arm prospective phase II study, first-line erlotinib and fixed-dose gemcitabine were evaluated in a population of previously untreated patients with locally advanced, inoperable, or metastatic PC. Although treatment was well tolerated, skin rash strongly predicted erlotinib efficacy, suggesting that a pharmacodynamic-based strategy for patient selection deserves further investigation [100]. Adding cetuximab to adjuvant gemcitabine does not seem to improve disease-free survival or OS of patients with unstratified PC [101].

Paclitaxel has wide applications in anti-cancer therapy, but was not previously considered an efficacious agent in PC. However, in the recently completed phase III MPACT trial, the addition of nab-paclitaxel to gemcitabine significantly improved the survival of patients with metastatic PC [102]. In the era of biological and molecularly targeted agents, the success of nab-paclitaxel in recalcitrant PC is a timely reminder of the importance and relevance of pharmacology and novel drug delivery technology in the development of PC drugs. In a phase I/II study of Chinese patients with previously untreated advanced PDAC, albumin-bound nab-paclitaxel (120 mg/m^2^), followed by gemcitabine (1000 mg/m^2^) administered intravenously for 30 min on days 1 and 8 and repeated every 21 days, had a favorable safety profile, with encouraging antitumor effects [103].

PEP02, also known as MM-398, is a novel nanoliposomal irinotecan that provides improved pharmacokinetics and tumor bio-distribution of the free drug. A phase II study evaluated PEP02 monotherapy as a second-line treatment in patients with metastatic PC that progressed following gemcitabine-based therapy. PEP02 demonstrated moderate antitumor activity with a manageable side effect profile for patients with metastatic, gemcitabine-refractory PC [104]. Another phase II study assessed the efficacy and safety of FOLFOX4 as a rescue therapy in patients for whom gemcitabine-based therapy failed. The objective response rate was 11.4%, and the tumor stabilization rate was 40.9%. The median time to progression was 9.9 weeks, and the median OS was 31.1 weeks [105]. In gemcitabine-refractory PC, FOLFOX4 showed encouraging activity and was generally well tolerated. However, careful attention must be paid to hematologic toxicities. Since capecitabine has theoretical pharmacokinetic advantages, such as higher intratumoral concentration and lower systemic concentration, retrospective analysis was performed to compare the outcomes, including toxicity, tumor response, and OS, of oral capecitabine plus radiotherapy (RT) with bolus 5-FU plus RT, in patients with locally advanced PC. Primary tumor and overall response rates and median OS time were similar in the two groups [106]. Capecitabine plus RT may be a safe and feasible regimen for patients with locally advanced PC, with similar efficacy to and lower toxicity than bolus 5-FU plus RT.

Long-term survival rates for patients with resected PC have been at 20% for more than a decade, illustrating the need to develop novel adjuvant therapies. Gemcitabine-erlotinib therapy has demonstrated a survival benefit for patients with metastatic PC. The first phase II study of erlotinib in combination with adjuvant chemoradiation and chemotherapy for resected PC showed a median recurrence-free survival of 15.6 months and a median OS of 24.4 months. Erlotinib can be administered safely with adjuvant IMRT-based CRT and chemotherapy [107]. The efficacy of this regimen appears comparable to that of existing adjuvant regimens.

The optimal treatment strategy for locally advanced and borderline resectable PC is unknown. Lloyd *et al.* compared three treatment strategies for OS: local control, metastasis-free survival, and surgical resection. Treatment with chemotherapy followed by chemoradiation therapy was associated with improved median OS compared with chemotherapy alone [108]. This strategy may select for patients who are less likely to develop early metastases and, therefore, have a better prognosis. Preoperative treatment with full-dose gemcitabine, oxaliplatin, and RT in patients with localized PC resulted in a high percentage of R0 resections [109]. These results are particularly encouraging because the majority of patients had borderline resectable disease. Five of 22 patients with locally advanced PC were able to undergo R0 resections following neoadjuvant FOLFIRINOX and chemoradiation. However, three of the five patients experienced distant recurrence within 5 months. FOLFIRINOX exhibited substantial activity in patients with LAPC, and was associated with conversion to resectability in >20% of patients [110]. However, the recurrence following R0 resection in some patients, and the toxicities observed with the use of this regimen, leave important questions about how best to treat such patients.

Metastatic PC carries a poor prognosis, with median survival on the order of several months. A multicenter phase II trial assessed the efficacy and toxicity of combined gemcitabine and candesartan therapy for advanced PC treatment. The overall response rate and disease control rates were 11.4% and 62.9%. The median PFS and OS were 4.3 and 9.1 months, with 1-year survival rate of 34.2%. The gemcitabine and candesartan combination therapy was tolerable, but failed to demonstrate activity against advanced PC. The median PFS was significantly longer in patients receiving higher doses of candesartan, suggesting that inhibition of the renin-angiotensin system may have a role in the treatment of advanced PC [111].

There is evidence that combining gemcitabine with either erlotinib or cisplatin may be superior to single-agent gemcitabine in patients with good performance. Khalil *et al.* retrospectively compared the outcomes of patients treated with either the three-drug regimen of gemcitabine, cisplatin, and erlotinib (GCE), or gemcitabine combined with cisplatin (GC), in order to assess the potential benefit of erlotinib. One-year survival was 23% in the GCE group, and 13% in the GC group [112]. Although there was a trend toward improved survival with GCE, the trend did not reach statistical significance, since the sample size was small.

A phase III study investigated the addition of aflibercept to gemcitabine in patients with advanced PC. Patients with metastatic PC received either i.v. aflibercept or placebo combined with gemcitabine. With a median follow-up of 7.9 months, based on the 546 patients at study termination, median OS was 7.8 months in the gemcitabine plus placebo arm (*n* = 275) *vs.* 6.5 months in the gemcitabine plus aflibercept arm (*n* = 271). This difference was not significant. Median PFS was 3.7 months in both arms [113]. In this study, the addition of aflibercept to gemcitabine did not improve OS in patients with metastatic PC.

In another phase II study of gemcitabine-pretreated advanced PC, Oxaliplatin Plus 5-Fluorouracil and Folinic Acid (OFF) gave a median duration of response of 13 weeks, with a median OS of 22 weeks and no treatment-related deaths. The 6-month survival rate was 30% [114]. This regimen is feasible and active with an acceptable toxicity; however, further phase III trials are needed.

A study to determine whether bolus regimens can replace FOLFOX for second-line treatment of advanced PC showed non-progression in 33.5% of those receiving FOLFOX treatment and in 29% of those receiving the bolus regimen. In addition, 37.5% had clinical benefit with the FLOX regimen, compared with 50% in the 3-week regimen. Both regimens showed acceptable toxicity and encouraging efficacy, with median OS of 8–9 months [115]. A toxicity study of gemcitabine, oxaliplatin, and bevacizumab, followed by 5-fluorouracil, oxaliplatin, bevacizumab, and radiotherapy in patients with locally advanced PC, showed higher response rates in those with metastatic disease, but a lower than expected response rate in the primary tumors [116]. Although tolerated by patients, this approach did not meaningfully affect clinical outcomes.

A study of gemcitabine plus capecitabine in 113 unstratified patients with advanced PC showed a median OS of 8.7 months, with 34% of patients alive one year after starting treatment [117].

A phase II randomized study of the urokinase inhibitor upamostat [WX-671; 200  mg (arm B) or 400  mg (arm C) daily PO] combined with i.v. gemcitabine compared with gemcitabine alone (arm A) in patients with non-resectable, locally advanced PC showed median OS of 12.5 months in arm C, 9.7 months in arm B, and 9.9 months in arm A. The corresponding 1-year survival rates were 50.6%, 40.7%, and 33.9%, respectively. More patients achieved a partial remission with upamostat combination therapy (arm C: 12.9%; arm B: 7.1%; arm A: 3.8%). Overall, only 12 patients developed detectable distant metastasis (arm A: 4, arm B: 6, arm C: 2). The most common adverse events considered to be related to upamostat were asthenia, fever, and nausea [118]. In this proof-of-concept study targeting the uPA (urokinase plasminogen activator) system in locally advanced PC, the addition of upamostat to gemcitabine was tolerated well.

Pharmacological doses of ascorbate, *i.e.*, ascorbic acid or vitamin C, was observed to be well tolerated when administered with gemcitabine in a phase 1 clinical trial [119]. However, further studies to determine efficacy are warranted.

In a phase II trial of erlotinib plus capecitabine as first-line treatment for metastatic PC (XELTA study), patients with untreated metastatic PC received oral capecitabine and oral erlotinib. The overall response rate (ORR) was 6%, with a median time to treatment failure of 2.1 months. The median PFS was 2.1 months; median OS was 4.3 months [120]. The combination of capecitabine with erlotinib might be an active regimen with a favorable safety profile for patients with metastatic PC.

In another study, lenalidomide combined with gemcitabine as first-line treatment for patients with previously untreated metastatic PDAC, with metastases incurable by surgery/radiation therapy, showed a 6-month OS of 37%, suggesting no improvement compared with historical results with gemcitabine alone [121]. Toxicities and dose modifications likely limited dose intensity. Further development of this regimen is not recommended for PC treatment.

Although FOLFIRINOX significantly increases survival in metastatic PC compared with gemcitabine, toxicities have tempered enthusiasm for its use in full doses. Of 35 patients, including those with locally advanced and metastatic PC, treated with this regimen, 29 received dose attenuations within the first cycle. Median relative doses of irinotecan and bolus fluorouracil were less than those reported previously. The response rate was 50% in locally advanced and 47% in metastatic PC (not significantly different). In patients with metastatic PC, OS at 6 and 12 months was comparable to the OS reported by others [122]. These findings validate the efficacy and tolerability of FOLFIRINOX in patients with locally advanced and metastatic PC, and suggest that dose attenuation of irinotecan and bolus fluorouracil might improve tolerability without compromising efficacy.

A recent meta-analysis of published trials determined that the addition of anti-EGFR drugs to gemcitabine-based chemotherapy improved OS compared with gemcitabine-based chemotherapy alone in patients with advanced PC. The addition of anti-VEGFR agents showed only a modest improvement in ORR, but not in PFS or OS [123]. FOLFIRINOX improved global health status of metastatic PC patients, but reduced quality of life (QoL) compared with the gemcitabine treatment arm [124]. In another phase I study of oxaliplatin in combination with gemcitabine, irinotecan, and 5-fluorouracil/leucovorin (G-FLIE) in patients with metastatic solid tumors, including PDAC, the overall median time to disease progression was 17 weeks, with median OS of 31.5 weeks. Except for one dose-limiting toxicity neutropenia and constipation, the MTD of oxaliplatin did not reach up to the pre-specified maximum level. Thus, G-FLIE is a tolerable multi-agent chemotherapy regimen with oxaliplatin doses of up to 85 mg/m^2^ [125]. The combination of full-dose oxaliplatin with gemcitabine, irinotecan, and 5-fluorouracil is feasible with attenuated doses of the drugs, but further optimization is necessary before efficacy can be assessed.

Overall, although combination chemotherapy has been employed extensively in patients with locally advanced or metastatic PC, and the outcomes from some clinical trials were encouraging, most regimens did not overcome toxicity issues or dramatically increase effectiveness. The chemoprevention aspects of combination regiments were not assessed in patients with PC due to the inability to diagnose patients at the very early stages of PC. Hence, to evaluate chemoprevention, we must examine multiple combinations and approaches using GEMs prior to assessment of the most effective combinations in clinical trials.

## 5. Dietary Strategies for the Prevention and Treatment of Pancreatic Cancer

**Figure 2:**
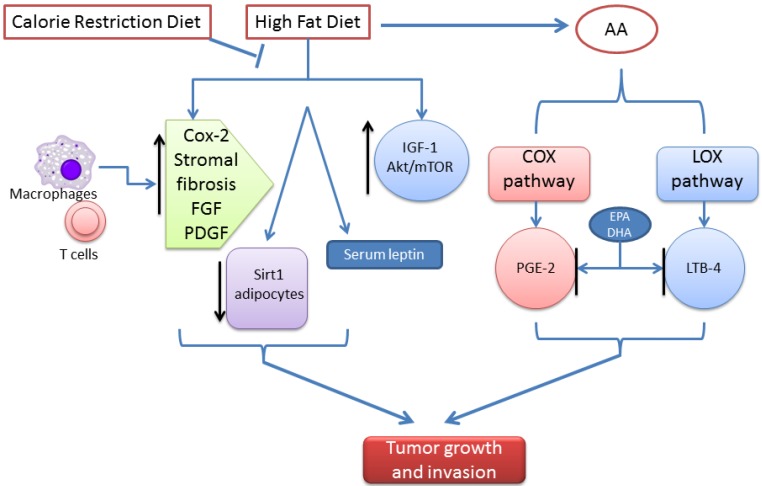
Dietary modulation influences the development of pancreatic cancer. High-fat diet containing PUFAs modulates pancreatic tumor development and progression. The PUFA arachidonic acid (AA; C20:4n-6) is the source of prostaglandin (PG)E2 and leukotriene (LT)B4, and promotes tumor growth and metastasis, whereas eicosanoids derived from the n-3 PUFAs eicosapentaenoic acid (EPA; C20:5n-3) and docosahexaenoic acid (DHA) show tumor-suppressive effects. Calorie restriction delays the progression of PC with decreased pmTOR and decreased serum IGF-1, decreased serum Insulin Growth Factor (IGF)-1, tumoral Akt/mTOR signaling, pancreatic desmoplasia, and progression to PDAC, and increased PC-free survival.

There is increasing evidence that dietary intake of n-3 fatty acids suppresses progression of cancers. A number of epidemiological and preclinical studies support a mostly protective effect of n-3 polyunsaturated fatty acids (PUFAs) against PC. Bioactive lipids containing PUFAs modulate a wide array of chronic diseases, including cancer development and progression. The PUFA arachidonic acid (AA; C20:4n-6) is the source of prostaglandin (PG)E_2_ and LTB_4_, which have been shown to promote tumor growth and metastasis, whereas eicosanoids derived from the n-3 PUFAs eicosapentaenoic acid (EPA; C20:5n-3) and docosahexaenoic acid (DHA) have tumor-suppressive effects [126,127,128]. Evidence suggests that the n6:n3 fatty acid (FA) ratio, rather than the absolute levels of these two classes of PUFAs, is the primary factor that influences tumorigenesis [129,130,131,132,133]. Recently, Strouch *et al.* showed that growth of EL-Kras mouse pancreatic tumors and human PC cell lines in high ω-3 FA concentrations mitigates pancreatic pre-cancer by inhibiting cell proliferation through induction of cell cycle arrest and apoptosis [134]. We recently demonstrated the effects of endogenous n-3 PUFAs derived from n-6 PUFAs on transgenic mice that exhibit a distinct PanIN-to-PDAC progression. The most striking finding was a dramatic inhibition of PDAC incidence and a decreased frequency of PanIN-3 formation and its progression to PDAC in Fat-1-p48^Cre/+^-LSL-Kras^G12D/+^ transgenic mice [135]. These results support the notion that increased tissue concentrations of omega-3 fatty acids can reduce pancreatic tumor progression. Additional research is needed to identify the molecular signaling pathways that are influenced by these dietary components, particularly FAs, and to help identify new drug targets. In LSL-Kras^G12D^/Pdx-1^Cre/Ink4a/Arflox/+^ mice, calorie restriction *vs.* obesity-inducing diet regimens decreased serum Insulin Growth Factor (IGF)-1, tumoral Akt/mTOR signaling, pancreatic desmoplasia, and progression to PDAC; and increased PC-free survival (Figure 2). Dietary energy balance modulation impacted spontaneous pancreatic tumorigenesis induced by mutant Kras and Ink4a deficiency, the most common genetic alterations in human PC [136]. These results suggest that IGF 1 and components of its downstream signaling pathway are promising targets for breaking the obesity-PC link.

Calorie restriction also delays the progression of preneoplastic lesions to PC in the LSL-Kras^G12D^; Pdx-1^Cre^ mouse model of PC. Mice fed ad libitum had a greater percentage of pancreatic ducts with PanIN-2 or -3 than did the intermittently calorie-restricted and chronically calorie-restricted groups. The delayed progression of lesions in calorie-restricted mice was associated with reduced proliferation, expression of the Glut1 glucose transporter, increased expression of Sirt1, increased serum adiponectin, and decreased serum leptin (Figure 2) [137]. Chronic calorie restriction resulted in decreased phosphorylated mTOR and decreased serum IGF-1. Administration of a calorie-restricted diet decreased serum IGF-1 levels, and hindered formation of pancreatic ductal lesions and dysplastic severity relative to a higher calorie control diet in transgenic mice overexpressing COX-2. These findings were correlated with reductions in Ki-67-positive cells, vascular luminal size, VEGF expression, and phosphorylation, and total expression of downstream mediators of the IGF-1 pathway [138]. These data suggest that modulation of this pathway with dietary and/or pharmacologic interventions is a promising PC prevention strategy.

Conversely, a high-fat diet was shown to activate oncogenic Kras and COX-2 to induce development of PDAC in mice. Mice with acinar cell-specific expression of Kras^G12D^ alone or progeny from a cross of a COX-2 conditional knockout mouse and LSL-Kras/Pdx1^Cre^ were fed isocaloric diets with different amounts of fat and a COX-2 inhibitor. Pancreatic tissues from mice fed the high-fat diets had increased Kras activity, fibrotic stroma, more PanINs and PDAC, and shorter survival times than mice fed control diets. Administration of a COX-2 inhibitor prevented the effects of the high-fat diet. The COX-2 knockout-Kras mice fed a high-fat diet had no evidence of increased numbers of PanIN lesions, inflammation, or fibrosis [139]. In mice, a high-fat diet can activate oncogenic Kras via COX-2, leading to pancreatic inflammation and fibrosis, and development of PanINs and PDAC. The role of a high-fat diet rich in saturated and n-6 PUFAs in the induction of inflammation and resistance to insulin is documented to be involved in increased risk of cancers, including PC. GEMs fed high-fat diet had higher body weights and increased inflammation (macrophages and T-cells), corresponding with an increase in high-grade PanIN lesions [140]. In a clinical study, the use of omega-3 fatty-acids-rich fish oil along with gemcitabine decreased inflammatory and angiogenic factors (interleukin-6 and interleukin-8) in patients with PC, leading to improved OS [141]. Further, one clinical study showed that patients with PC given approximately 2 g of EPA and 1 g of DHA per day for 7 weeks showed significant weight gain and improvements in functional status and appetite [142].

## 6. Phytochemicals for Pancreatic Cancer Prevention and Treatment

A comprehensive analysis of 200 published articles provided a strong correlation between the consumption of fruits and vegetables and reduced incidence of cancers, including PC. The advantages of bioactive dietary agents, or nutraceuticals, are increased cost effectiveness and reduced toxicity. Some of the phytochemicals that have promising activity against PC are curcumin, capsaicin, triterpenoids, resveratrol, and green tea.

Phase I clinical trials with curcumin have shown that curcumin is relatively safe, even at a high dose of 12 g/day in humans. However, curcumin has limited bioavailability because of poor absorption, rapid metabolism, and rapid systemic elimination [143]. Phase I/II clinical trials in patients with gemcitabine-resistant PC showed that oral administration of 8 g of curcumin resulted in an increased median survival time (MST) of 161 days, and a one-year survival rate of 19% [144]. Efforts are underway to increase the bioavailability of curcumin by various approaches. The curcumin analogue difluorinated-curcumin (CDF) showed better bioavailability and 10-fold higher concentrations in the pancreas compared with curcumin [145]. In another phase II clinical trial of 25 chemotherapy patients receiving 8 g/day curcumin, two patients demonstrated clinical benefit and one patient had stable disease for 18 months [146]. Repetitive systemic exposure to high concentrations of Theracurmin®, a new form of curcumin developed to increase bioavailability, did not increase the incidence of adverse events in patients with cancer who were receiving gemcitabine-based chemotherapy [147]. We observed a ~50% inhibition of PDAC incidence in p48^Cre/+^-LSL-Kras^G12D/+^ transgenic mice with curcumin (unpublished data). Although curcumin may be a potential anticancer agent, further studies are needed to find ways to increase its bioavailability.

Capsaicin is a major biologically active ingredient of chili peppers. LSL-Kras^G12D^/Pdx1^Cre^ (KPC) mice fed a daily diet including 10 or 20 ppm of capsaicin for eight weeks had significantly reduced severity of caerulin-induced chronic pancreatitis and PanIN lesions [148]. As mentioned above, the non-cytotoxic synthetic oleanane triterpenoids (CDDO-Me and CDD-ethylamide) significantly (*p* < 0.05) increased survival in KPC mice by 3-to-4 weeks when combined with the rexinoid LG100268 [84]. Resveratrol is a naturally occurring polyphenolic phytochemical found in many plant species, including grapes, peanuts, and herbs. Resveratrol inhibited the development and growth (size and weight) of PanIN lesions in Kras^G12D^ mice. Resveratrol also inhibited the self-renewal capacity of pancreatic cancer stem cells (CSCs) derived from human primary tumors and Kras^G12D^ mice. These data suggest that resveratrol inhibits PC stem cell characteristics in humans and Kras^G12D^ transgenic mice by inhibiting EMT and factors that maintain pluripotency [149].

The association of tea drinking with the risk of developing PC was examined in a population-based, case-controlled study conducted in urban Shanghai, comparing 908 cases of PC with 1067 healthy controls. In women, regular green tea drinking was associated with a 32% reduction of PC risk, *OR* = 0.68, 95% CI [0.48–0.96]. A reduced PC risk was seen in the women who consumed tea for longer durations. These results show that the habit of green tea drinking, including regular drinking, amount of consumption, and persistence of the habit, may lower PC risk [150]. In a placebo-controlled, double-blind clinical trial involving patients undergoing pancreatic duodenectomy, the impact of a preconditioning oral nutritional supplement enriched with glutamine, green tea extract, and antioxidants was evaluated on post-operative oxidative stress. Plasma vitamin C levels were positively affected by perioperative pONS administration, but it did not reduce the oxidative stress and systemic inflammation markers [151].

## 7. Immuno-Chemoprevention and Immunotherapy for Pancreatic Cancer

**Figure 3:**
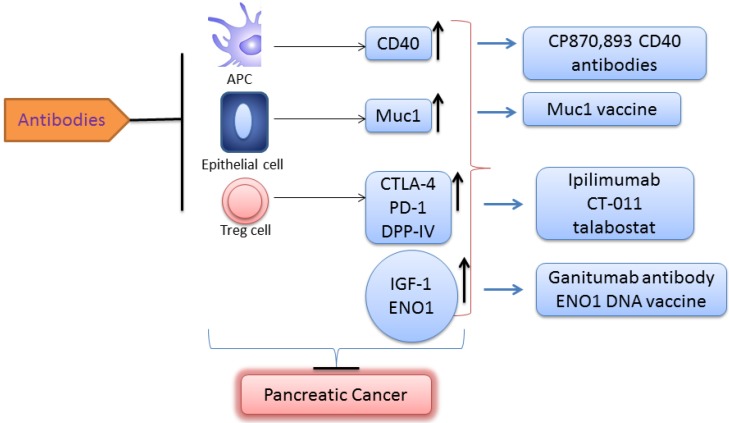
Developing vaccines for pancreatic cancer treatment. Antibodies/vaccines CP870,893, Muc1 vaccine, Ipilimumab, CT-011, and talabostat regulate the responses of immune modulatory cells by targeting their signaling molecules, including CD40, MUC1, cytotoxic T lymphocyte associated antigen-4 (CTLA-4), programmed Death-1 receptor (PD-1), and DPP-IV, respectively. CD40, expressed on antigen-presenting cells, mediates tumor-specific priming and expansion of T lymphocytes. CTLA-4 modulates activation of T cells through regulation of T cell receptor/CD28 signaling. PD-1 is expressed on activated T lymphocytes and is involved in immune suppression. Mucin-1 (MUC1) is a membrane-bound hypoglycosylated phosphoprotein overexpressed in pancreatic tumors. Antibodies against α-enolase (ENO1), a glycolytic enzyme, are detected in patients with PDA and ENO1-specific T cells. The ENO1 vaccine induces antibodies and a cellular response, increases survival time, and appears to slow tumor progression. Vaccination with ENO1 DNA elicited humoral and cellular immune responses against tumors, delayed tumor progression, and significantly extended survival in mice. A randomized phase II study of ganitumab (monoclonal antibody inhibitor of IGF-1 receptor) plus gemcitabine in metastatic PC revealed improved OS in patients with higher levels of IGF-1, IGF-2, or IGFBP-3, or lower levels of IGFBP-2.

Most patients exhibit tumor-specific cellular immunity to their cancer antigens. However, tumors become immune-tolerant as the tumor microenvironment adopts multiple ways to resist death by immune effector cells [152]. Unfortunately, neither PC vaccines nor therapeutic drugs are efficacious in advanced stage cancers, because of lowered immunity. More than 90 clinical trials have tested therapeutic vaccines in patients with different cancers, but the clinical benefits are modest. Recent attempts have been made to regulate the responses of various immune modulatory cells by targeting their signaling molecules, including CD40, MUC1, cytotoxic T lymphocyte associated antigen-4 (CTLA-4), programmed Death-1 receptor (PD-1), and Dipeptidyl peptidase-IV (DPP-IV). CD40, expressed on antigen-presenting cells, mediates tumor-specific priming and expansion of T lymphocytes. A combination of a monoclonal antibody (CP-870,893) against CD40 with gemcitabine induced a measurable anti-tumor immune response (Figure 3) [153]. In GEM models, CD40 antibodies caused tumor regression by inducing macrophage infiltration into the tumors and degradation of tumor-associated stroma [153]. CTLA-4 modulates activation of T cells through regulation of T cell receptor/CD28 signaling [154,155]. Ipilimumab, an antibody that binds to CTLA4, causes augmentation of T-cell activation and proliferation. However, no response was observed in a phase II trial of ipilimumab [156]. PD-1 is expressed on activated T lymphocytes and is involved in immune suppression [157]. An ongoing phase II clinical trial with CT-011, a recombinant monoclonal antibody directed against the PD-1 receptor, seeks to determine safety and efficacy. Similarly, talabostat, a competitive inhibitor of DPP-IV, is being investigated in metastatic PC (Figure 3).

Mucin-1 (MUC1) is a membrane-bound hypoglycosylated phosphoprotein that is overexpressed in pancreatic tumors. Muc1 has been extensively studied in both preclinical and clinical tissues (Figure 3) [158]. Muc1 overexpression leads to heavy glycosylation in the extracellular domain of MUC1 in PC. The glycosylation blocks access of chemotherapeutic drugs to cancer cells and enriches growth factors near their receptors, thus increasing receptor activity and cancer cell growth. In addition, MUC1 prevents the interaction between immune cells and receptors on the cancer cell surface through steric hindrance. Evidence shows that Muc1 interacts with p53, EGFR, and the activation of p-Akt/mTOR signaling pathways [159]. Thus, Muc1 is considered a valid target for cancer treatment. Immune responses to a Muc1 vaccine (100-amino acid peptide corresponding to five 20-amino-acid-long repeats) have been tested in preclinical and clinical studies [62,160,161,162,163,164,165]. There were no adverse side effects when the Muc1 vaccine was given with adjuvant therapy [160]. In a phase I/II study of a Muc1 peptide-pulsed autologous dendritic cell vaccine as adjuvant therapy in patients with resected pancreatic and biliary tumors, four of twelve patients lived for over four years [164]. Muc1 overexpression is associated with early PanIN lesions. Thus, development of Muc1 vaccines for PC prevention is a valid approach. Mukherjee *et al.* showed that a MUC1-based vaccine combined with a COX-2 inhibitor (celecoxib) and low-dose gemcitabine was effective in preventing or delaying the progression of PanIN lesions to invasive PDAC in p48^Cre/+^-LSL-Kras^G12D/+^ transgenic mice that express the human Muc1 gene. Robust antitumor cellular and humoral immune responses were elicited by the combination treatment, and were associated with increased apoptosis in the tumor. The increased immune response was attributed to decreased levels of T regulatory and myeloid suppressor cells within the tumor microenvironment, and downregulation of circulating prostaglandin E2 and indoleamine 2,3-dioxygenase enzymatic activity [62]. These preclinical studies provide the rationale for clinical trials with a combination of MUC1-based vaccines and anti-inflammatory agents for the prevention and treatment of PC.

Capello *et al.* demonstrated that the α-enolase (ENO1) DNA vaccine induced immune responses, increased survival of GEMs (Figure 3), and reduced T-regs, with increased Th1 and Th17 immune responses [165]. Gemcitabine became the reference regimen for advanced PC after a randomized trial showed a significant improvement in median OS after treatment. Since then, the combination of gemcitabine with a variety of agents has generally shown no significant survival advantage compared with gemcitabine alone. In one study, a GEM model of PC was used to evaluate the chemotherapeutic potential of aspirin as an adjuvant agent to gemcitabine. Gemcitabine prolonged the median OS of transgenic mice. The addition of aspirin to gemcitabine extended survival for ten more days [166]. Administration of aspirin in combination with gemcitabine significantly reduced the number of Foxp3+ regulatory T cells. Thus, aspirin may be effective for use with gemcitabine in the treatment of PC. Further studies are needed to confirm whether aspirin may be a well-tolerated chemotherapeutic adjuvant agent for PC.

Liu *et al.* studied the therapeutic efficacy and mechanisms of action of herpes simplex virus encoding granulocyte-macrophage colony-stimulating factor (HSVGM-CSF) in PC. Administration of recombinant mouse HSVGM-CSF resulted in a significant dose-dependent reduction in tumor growth, and the production of GM-CSF was significantly increased. This study provides the first evidence that an HSVGM-CSF vaccine could inhibit PC growth [167].

A phase I study investigated the maximum tolerated dose (MTD), safety, pharmacodynamics, immunological correlatives, and anti-tumor activity of CP-870,893, a CD40 agonist antibody, when administered in combination with gemcitabine in patients with advanced PDAC. The combination was well-tolerated and associated with anti-tumor activity [168]. Changes in fluoro-deoxyglucose (FDG) uptake detected on PET/CT imaging provided some insight into therapeutic benefit. However, further studies are warranted.

Patients with PDAC were treated with ipilimumab, *i.e.*, anti-CTLA-4, alone or combined with GVAX, to determine whether the combined vaccine and checkpoint blockade offer advantages. The combination increased median survival. Thus, this combined treatment has the potential for clinical benefit and should be evaluated in a larger study [169].

Regulatory T cells (Tregs) play a role in immunosuppression. Tregs were significantly reduced after gemcitabine chemotherapy. Other immune cells were examined; the proliferative capacity did not change [170]. These researchers argued that gemcitabine-based chemotherapy produced an immunomodulatory effect via the depletion of Tregs. A phase II trial using peptide vaccine at personalized level (PPV) for patients with advanced, chemotherapy-resistant PC showed that high levels of serum amyloid A and c-reactive protein are detrimental to overall survival [171]. The Japanese Kampo medicine Juzen-Taihoto/TJ-48 is empirically considered to be an immunoaugumentation drug. Peripheral administration of Juzen-Taihoto/TJ-48 to patients with advanced PC significantly decreased Foxp3(+) Treg populations and increased the CD4/CD8 ratio, even though CD57(+) NK cell populations did not significantly change [172]. This effect could lead to immunoaugumentation for various combination therapies.

Despite continued investigation, limited progress has been made in adjuvant treatment for resected PC. In a multi-institutional, open-label, dose-finding, phase II trial evaluating the use of algenpantucel-L immunotherapy in addition to chemotherapy and chemoradiotherapy in the adjuvant setting for resected PC, patients were treated with gemcitabine and 5-fluorouracil-based chemoradiotherapy, and algenpantucel-L. After a median follow-up of 21 months, the 12-month disease-free survival was 62%, and the 12-month OS was 86%, suggesting that the addition of algenpantucel-L to standard adjuvant therapy for resected PC may improve survival. The most common adverse events were injection site pain and induration [173]. A multi-institutional, phase 3 study is ongoing.

In a combination treatment with comprehensive cryoablation, immunotherapy, and/or chemotherapy in metastatic PC, the median OS was higher in the cryoimmunotherapy and cryotherapy groups than in the chemotherapy group and was higher in the cryoimmunotherapy group than in the cryotherapy and immunotherapy groups. Cryoimmunotherapy significantly increased OS in metastatic PC [174].

The predictive nature of baseline circulating components of the IGF axis on the treatment effect of ganitumab plus gemcitabine was assessed in a randomized phase II study in metastatic PC [175]. OS was higher in the patient subsets with higher levels of IGF-1, IGF-2, or IGFBP-3, or lower levels of IGFBP-2. In patients with higher levels of IGF-1, IGF-2, and IGFBP-3, median OS with ganitumab *vs.* placebo was 16 *vs.* 6.8 months, 16 *vs.* 5.9 months, and 16 *vs.* 6.8 months. In patients with lower IGFBP-2 levels, median OS was 12.7 months (ganitumab) *vs.* 6.6 months (placebo). The interaction between treatment and IGFs/IGFBPs in multivariate analyses suggested predictive potential for IGF-2 and IGFBP-2.

## 8. Other Novel Agents for Pancreatic Cancer Prevention and Treatment

Several studies have used GEM models of PC to target other important signaling components (Table 1), such as JNK, nitric oxide synthase (NOS), VEGF, ACE, gamma-secretase, and Hedgehog signaling, in an attempt to delay disease progression. JNK is frequently activated in human and murine PC. Treatment of Ptf1a^Cre/+^; LSL-Kras^G12D/+^; Tgfbr2^flox/flox^ mice with a JNK inhibitor decreased growth of murine PC, inhibited angiogenesis and prolonged survival of the mice [176]. Minnelide, a water-soluble analog of triptolide, was highly effective in reducing PC growth and spread and improving survival in Pdx1^Cre^.LSL-Kras^G12D^.Trp53^R172H^ mice [177]. The activation of endothelial nitric oxide synthase (eNOS or NOS III) has been recently implicated in the pathogenesis of PDAC. The NOS small molecule inhibitor (*L*-NAME) was found to inhibit tumor growth [178]. Anti-VEGF antibodies, alone or in combination with gemcitabine or erlotinib, inhibited disease progression [179,180]. Aspirin and ACE inhibitors, such as enalapril, were found to be effective chemopreventive agents, by delaying progression of PanINs and partially inhibiting the formation of PC in transgenic Kras mice [83].

Notch signaling is required for PDAC progression. The effects of a gamma-secretase inhibitor (GSI) that blocks Notch signaling in PDAC cell lines and in a GEM model of PDAC (Pdx1^Cre^.LSL-Kras^G12D^.Trp53^R172H^ mice) suggest that pharmacologic targeting of this pathway has therapeutic potential in this treatment-refractory malignancy [181]. Hedgehog inhibition with cyclopamine significantly prolonged median survival to 67 days *vs.* 61 days (*p* = 0.026) in the transgenic mouse model [182]. The PI3K inhibitor GDC 0941 effectively blocked tumor growth in transgenic Kras mice [183]. The ornithine decarboxylase inhibitor DFMO (eflornithine) significantly inhibited the progression of PanIN lesions to carcinoma in Kras transgenic mice [184].

SRC kinases that are controlled by several kinase receptors, like EGFR, FGFR, and G-protein coupled receptors, are known to be overexpressed in several cancers, including PC. In this direction, SRC inhibitor saracatinib (AZD0530) was evaluated in combination with gemcitabine in a phase I/II clinical trial [185]. Although the combination treatment was well tolerated, it did not improved overall efficacy [185].

## 9. Tumor Microenvironment-Targeted Strategies for Pancreatic Cancer Prevention and Treatment

**Figure 4:**
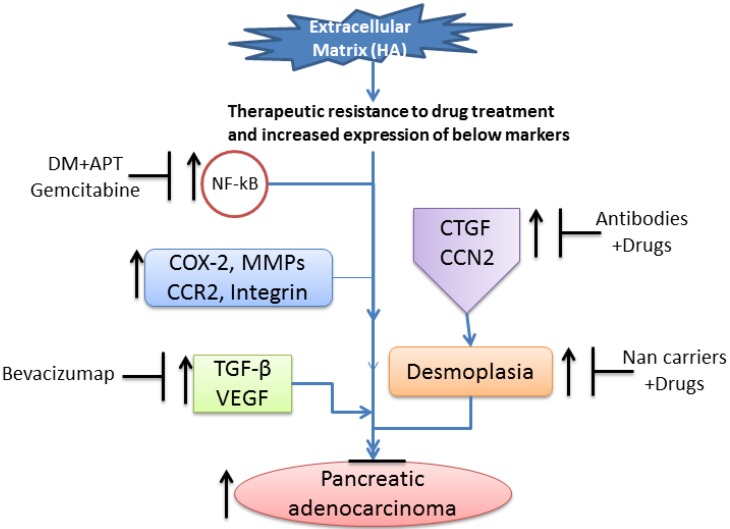
Tumor microenvironment-based treatment strategies for PC. PC is characterized by a stromal reaction with marked fibrosis (desmoplasia). Tumor stroma is a complex microenvironment comprised of extracellular matrix (ECM), activated fibroblasts, immune cells, inflammatory cells, and aberrant vasculatures. The vasculature deficiency in the matrix is a contributing factor to chemoresistance and radiotherapeutic resistance, impeding drug delivery. The components of the tumor microenvironment, NFkβ, COX-2, MMPs, CCR2, integrin, TGFβ, CTGF, CCN2, and desmoplasia, induce therapeutic resistance and aggravate pancreatic carcinoma. Tumor microenvironment-based strategies, such as DMAPT + gemcitabine for NFkβ, bevacizumab for VEGF and TGF-β, a combination of antibodies with drugs for CTGF and CCN2, and a combination of nanocarriers with drugs for desmoplasia, have been investigated as treatment strategies for PC. In combination with standard chemotherapy drug gemcitabine, these treatments permanently remodel the tumor microenvironment and consistently achieve objective tumor response. Targeting the tumor microenvironment may be a valid approach for PC prevention and treatment.

PDAC is characterized by a stromal reaction with marked fibrosis (desmoplasia). Tumor stroma is a complex microenvironment comprised of extracellular matrix (ECM), activated fibroblasts, immune cells, inflammatory cells, and aberrant vasculatures. The ECM component hyaluronic acid (HA), or hyaluronan, is inordinately abundant, leading to speculation regarding its role in disease resistance. The vasculature deficiency in the matrix is a contributing factor to chemoresistance and radiotherapeutic resistance by impeding drug delivery. Several of the components (SPARC, MMPs, COX-2, VEGF, CCR2, integrin) of tumor-associated stroma have been investigated as therapeutic targets for PC (Figure 4).

A phase I/II trial using a combination of SPARC-regulating nab-paclitaxel and gemcitabine showed remarkable response and patient survival rates. The MMP inhibitor marimastat showed no significant difference in OS in a Phase II clinical trial. BAY 12-9566, another MMP inhibitor, was observed to be inferior to gemcitabine. Several clinical trials of bevacizumab, a monoclonal antibody that inhibits neovascularization and tumor growth, alone or in combination with other drugs, are ongoing or completed. The tumor tissues in experimental mouse models of PC showed strong expression of connective tissue growth factor (CTGF), a profibrotic and tumor-promoting factor, especially in the tumor–stromal border area. Inhibiting tumor-stromal interactions could be exploited as a therapeutic strategy [83]. A recent study has shown that enzymatic targeting of the stroma eliminates physical barriers to treatment of PDAC. Hence, targeting the tumor microenvironment may be a valid approach for the prevention and treatment of PC.

Two cycles of nab-paclitaxel and gemcitabine resulted in significant antitumor effects before surgical resection in patients with operable PDAC, with 50% of patients achieving a >75% decrease in the circulating carbohydrate-associated antigen (CA) 19.9 tumor marker. Analysis of residual tumors showed markedly disorganized collagen. A preclinical study in a mouse model of PDAC showed that these effects were specific to nab-paclitaxel, and not gemcitabine [186]. These data suggest that nab-paclitaxel and gemcitabine decrease CAF (cancer-associated fibroblasts) content and induce a marked alteration in the cancer stroma that results in tumor softening. This regimen could be studied in patients with operable PDAC.

In order not to disturb the tumor microenvironment, but to target cytidine deaminase and enhance the availability of gemcitabine in tumors in GEMs, Neesse *et al.* used cytidine deaminase inhibitor, alone or in combination with FG-3019 antibody. Treatment resulted in increased levels of gemcitabine in tumors [187]. Nanoscaled drug-loaded carriers are of particular interest for efficient tumor therapy, as numerous studies have shown improved targeting and efficacy with this delivery method. However, most of these studies have been performed in allograft and xenograft tumor models, which have altered microenvironment features, affecting the accumulation and penetration of nanocarriers. The evaluation of nanocarriers in GEMs, which can gradually develop clinically relevant tumors, permits validation of their design under normal processes of immunity, angiogenesis, and inflammation. Therefore, considering the poor prognosis of PC, the elastase 1-promoted luciferase and Simian Virus 40 T and transgenic mice that develop spontaneous bioluminescent PC were used. Long circulating micellar nanocarriers incorporating the parent complex of oxaliplatin inhibit tumor growth as a result of their efficient accumulation in the tumors. The reduction of the photon flux from the endogenous tumor in response to the micelles was correlated with the decrease of the serum CA19-9 marker. Micelles also reduced the incidence of metastasis and ascites, extending survival [188].

The utility of DMAPT (dimethylaminoparthenolide), individually and in combination with gemcitabine, for PC prevention was evaluated using Kras and P53-expressing GEMs. The combination treatment significantly decreased tumor growth and liver metastasis, and reduced inflammatory cytokines TNFα and NF-κB (Figure 4) [189]. Similarly, TNFα blocker etanercept was studied with gemcitabine in a phase II trial with patients with advanced PC. The combination was found to be safe, but failed to show synergism [190].

Serum pro-inflammatory cytokines were evaluated before and after gemcitabine chemotherapy. High IL-6 and IL-1β levels were poor prognostic factors for OS. Patients with both high IL-6 and high IL-1β exhibited shortened OS and PFS, a reduction in the tumor control rate, and a high dose intensity of gemcitabine, compared with patients with low levels of both IL-6 and IL-1β [191]. Hence, the pre-treatment serum levels of IL-6 and IL-1β predict the efficacy of gemcitabine in patients with advanced PC.

## 10. Conclusions and Prospective

At present, there are no curative therapies for patients with PC, due to the lack of specific early-stage clinical symptoms and the highly aggressive nature of the evolving disease. Consequently, the average survival of resected patients is approximately 12-to-20 months, with a high probability of relapse. Use of gemcitabine alone or in combination with other agents has demonstrated somewhat enhanced efficacy in the treatment of PC.

**Figure 5:**
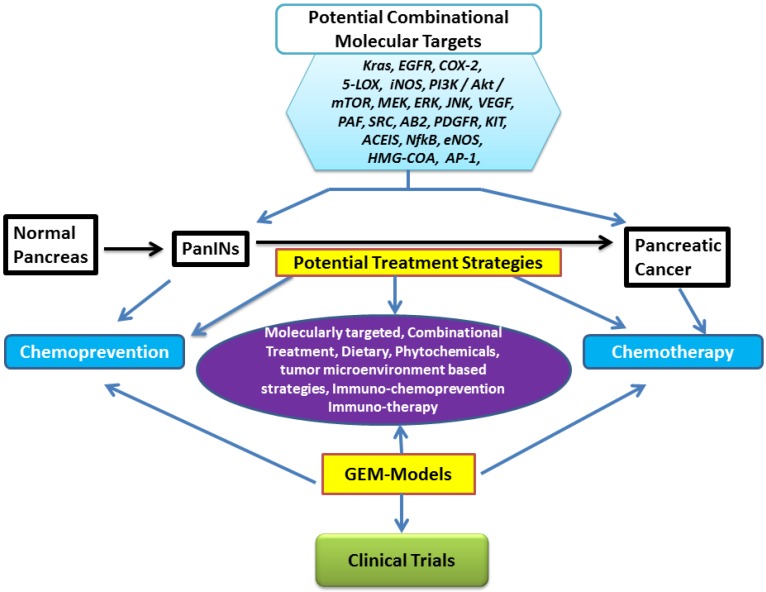
The integration of knowledge gained from scientific advances in pancreatic cancer research. The Kras mutation in the pancreas leads to the formation and progression of PanIN lesions to pancreatic ductal adenocarcinoma. Potential multiple molecular alterations, including Kras, EGFR, COX-2, 5-LOX, iNOS, PI3K/AKT/mTOR, MEK, ERK, JNK, VEGF, PAF, SRC, AB2, PDGFR, KIT, ACEIS, NFkβ, eNOS, HMG-COA, and AP-1, occur during the progression of PanIN lesions. Chemoprevention can be employed at the PanIN stage, whereas chemotherapy is employed at the PC stage by targeting individual or combined treatment strategies, dietary, phytochemical, tumor microenvironment-based strategies, immune-chemoprevention, or immune-chemotherapy, using preclinical GEM models or clinical trials.

Despite these advances, PC remains one of the most deadly malignancies. Approved clinical therapies and ongoing clinical trials with new drugs, vaccines, or combinations have provided modest survival improvement. The main reasons for lack of effective treatment for this deadly disease relate to the complexity of cancer cells, with accumulating mutations in multiple signaling pathways, and a multifaceted tumor microenvironment. Knowledge of the molecular genetics of PC carcinogenesis generated with the advent of humanized GEM models is beginning to be translated into therapeutic and chemopreventive strategies, including development of agents and vaccines directed against multiple targets. The use of GEM models has led researchers to discover genetic and epigenetic regulation of various biological processes, and to generate new insights into how the regulation becomes aberrant and leads to initiation of pancreatic neoplasia and progression to adenocarcinoma. Recent studies with these models suggest that the following may be effective strategies for PC prevention and treatment: (1) targeting multiple signaling molecules with combinations of individual drugs; (2) use of a few agents that target multiple signaling molecules; or (3) combinations of vaccines with immuno-modulatory drugs. Preclinical studies using GEM models are expected to facilitate the investigation of the molecular mechanisms of PC and the testing and validation of treatment and prevention strategies. As shown in Figure 5, integration of the available knowledge of PC should help with the development of new diagnostic screens and with the design of improved prevention and treatment strategies.

## References

[1] American Cancer Society (2015). Cancer Facts and Figures 2015.

[2] Burris H.A., Moore M.J., Andersen J., Green M.R., Rothenberg M.L., Modiano M.R., Cripps M.C., Portenoy R.K., Storniolo A.M., Tarassoff P. (1997). Improvements in survival and clinical benefit with gemcitabine as first-line therapy for patients with advanced pancreas cancer: A randomized trial. J. Clin. Oncol..

[3] Heinemann V. (2001). Gemcitabine: Progress in the treatment of pancreatic cancer. Oncology.

[4] Mamon H.J., Niedzwiecki D., Hollis D., Tan B.R., Mayer R.J., Tepper J.E., Goldberg R.M., Blackstock A.W., Fuchs C.S. (2011). A phase 2 trial of gemcitabine, 5-florouracil, and radiation therapy in locally advanced nonmetastatic pancreatic adenocarcinoma: Cancer and Leukemia Group B (CALGB) 80003. Cancer.

[5] Kim M.P., Gallick G.E. (2008). Gemcitabine resistance in pancreatic cancer: Picking the key players. Clin. Cancer Res..

[6] Hidalgo M. (2010). Pancreatic cancer. N. Engl. J. Med..

[7] Eckel F., Schneider G., Schmid R.M. (2006). Pancreatic cancer: A review of recent advances. Exp. Opin. Investig. Drugs.

[8] Mohammed A., Janakiram N.B., Lightfoot S., Gali H., Vibhudutta A., Rao C.V. (2012). Early Detection and Prevention of Pancreatic Cancer: Use of Genetically Engineered Mouse Models and advanced Imaging Technologies. Curr. Med. Chem..

[9] Sayers H.J., Orloff M.J. (1976). Development of an animal model of pancreatic cancer. Surg. Forum.

[10] Morosco G.J., Goeringer G.C. (1980). Lifestyle factors and cancer of the pancreas: A hypothetical mechanism. Med. Hypotheses.

[11] Berlin N.I. (1981). Williams M. Pancreatic cancer: An epidemiologic approach and model. J. Am. Med. Assoc..

[12] Hruban R.H., Goggins M., Parsons J., Kern S.E. (2000). Progression model for pancreatic cancer. Clin. Cancer Res..

[13] Mazur P.K., Siveke J.T. (2012). Genetically Engineered mouse models of pancreatic cancer: Unravelling tumor biology and progressing translational oncology. Gut.

[14] Mihaljevic A.L., Michalski C.W., Friess H., Kleeff J. (2010). Molecular mechanism of pancreatic cancer—Understanding proliferation, invasion, and metastasis. Langenbecks Arch. Surg..

[15] Hong S.M., Park J.Y., Hruban R.H., Goggins M. (2011). Molecular Signatures of Pancreatic Cancer. Arch. Pathol. Lab. Med..

[16] Feldmann G., Beaty R., Hruban R.H., Maitra A. (2007). Molecular genetics of pancreatic intraepithelial neoplasia. J. Hepato-Biliary-Pancreat Surg..

[17] Hilgers W., Kern S.E. (1999). Molecular Genetic Basis of Pancreatic Adenocarcinoma. Genes Chromosom. Cancer.

[18] Feldmann G., Habbe N., Dhara S., Bisht S., Alvarez H., Fendrich V., Beaty R., Mullendore M., Karikari C., Bardeesy N. (2008). Hedgehog inhibition prolongs survival in a genetically engineered mouse model of pancreatic cancer. Gut.

[19] Ottenhof N.A., Milne A.N., Morsink F.H., Drillenburg P., Ten Kate F.J., Maitra A., Offerhaus G.J. (2009). Pancreatic Intraepithelial Neoplasia and Pancreatic Tumorigenesis of Mice and Men. Arch. Pathol. Lab. Med..

[20] Jones S., Zhang X., Parsons D.W., Lin J.C., Leary R.J., Angenendt P., Mankoo P., Carter H., Kamiyama H., Jimeno A. (2008). Core signaling pathways in human pancreatic cancers revealed by global genomic analyses. Science.

[21] Haas M., Laubender R.P., Stieber P., Holdenrieder S., Bruns C.J., Wilkowski R., Mansmann U., Heinemann V., Boeck S. (2010). Prognostic relevance of CA 19-9, CEA, CRP, and LDH kinetics in patients treated with palliative second-line therapy for advanced pancreatic cancer. Tumour Biol..

[22] Ho J.J., Siddiki B., Kim Y.S. (1995). Association of sialyl-Lewis(a) and sialyl-Lewis(x) with MUC-1 apomucin ina pancreatic cancer cell line. Cancer Res..

[23] Steinberg W. (1990). The clinical utility of the CA 19-9 tumor-associated antigen. Am. J. Gastroenterol..

[24] Andren-Sandberg A. (1989). CA 50 and CA 19-9 in serum as tumor markers for pancreatic cancer: A review of the literature. Acta Chir. Scand..

[25] Kokhanenko N., Ignashov A.M., Varga E.V., Polkanova M.S., Aleshina L.A., Kimbarovskaia A.A., Osipenko S.K., Lebedev E.G. (2001). Role of the tumor markers CA 19-9 and carcinoembryonic antigen (CEA) in diagnosis, treatment and prognosis of pancreatic cancer. Vopr. Onkol..

[26] Baeckstrom D., Hansson G.C., Nilsson O., Johansson C., Gendler S.J., Lindholm L. (1991). Purification and characterization of a membrane-bound and a secreted mucin-type glycoprotein carrying the carcinoma-associated sialyl-Lea epitope on distinct core proteins. J. Biol. Chem..

[27] Kelly K.A., Bardeesy N., Anbazhagan R., Gurumurthy S., Berger J., Alencar H., Depinho R.A., Mahmood U., Weissleder R. (2008). Targeted Nanoparticles for Imaging Incipient Pancreatic Ductal Adenocarcinoma. PLoS Med..

[28] Haug U., Wente M.N., Seiler C.M., Jesenofsky R., Brenner H. (2008). Stool testing for the early detection of pancreatic cancer: Rationale and current evidence. Exp. Rev. Mol. Diagn..

[29] Hingorani S.R., Wang L., Multani A.S., Combs C., Deramaudt T.B., Hruban R.H., Rustgi A.K., Chang S., Tuveson D.A. (2005). Trp53R172H and KrasG12D cooperate to promote chromosomal instability and widely metastatic pancreatic ductal adenocarcinoma in mice. Cancer Cell.

[30] Izeradjene K., Combs C., Best M., Gopinathan A., Wagner A., Grady W.M., Deng C.X., Hruban R.H., Adsay N.V., Tuveson D.A. (2007). KrasG12D and Smad4/Dpc4 Haploinsufficiency Cooperate to Induce Mucinous Cystic Neoplasms and Invasive Adenocarcinoma of the Pancreas. Cancer Cell.

[31] Shi G., Zhu L., Sun Y., Bettencourt R., Damsz B., Hruban R.H., Konieczny S.F. (2009). Loss of the Acinar-Restricted Transcription Factor Mist1 Accelerates Kras-Induced Pancreatic Intraepithelial Neoplasia. Gastroenterology.

[32] Tuveson D.A., Zhu L., Gopinathan A., Willis N.A., Kachatrian L., Grochow R., Pin C.L., Mitin N.Y., Taparowsky E.J., Gimotty P.A. (2006). Mist1-KrasG12D Knock-In Mice Develop Mixed Differentiation metastatic Exocrine Pancreatic Carcinoma and Hepatocellular Carcinoma. Cancer Res..

[33] Grippo P.J., Nowlin P.S., Demeure M.J., Longnecker D.S., Sandgren E.P. (2003). Preinvasive pancreatic neoplasia of ductal phenotype induced by acinar cell targeting of mutant Kras in transgenic mice. Cancer Res..

[34] Hingorani S.R., Petricoin E., Maitra A., Rajapakse V., King C., Jacobetz M.A., Ross S., Conrads T.P., Veenstra T.D., Hitt B.A. (2003). Preinvasive and invasive ductal pancreatic cancer and its early detection in the mouse. Cancer Cell.

[35] Yachida S., Jones S., Bozic I., Antal T., Leary R., Fu B., Kamiyama M., Hruban R.H., Eshleman J.R., Nowak M.A. (2010). Distant metastasis occurs late during the genetic evolution of pancreatic cancer. Nature.

[36] Moore M., Goldstein D., Hamm J., Figer A., Hecht J.R., Gallinger S., Au H.J., Murawa P., Walde D., Wolff R.A. (2007). Erlotinib plus gemcitabine compared with gemcitabine alone in patients with advanced pancreatic cancer: A phase III trial of the National Cancer Institute of Canada Clinical Trials Group. J. Clin. Oncol..

[37] Conroy T., Desseigne F., Ychou M., Bouché O., Guimbaud R., Bécouarn Y., Adenis A., Raoul J.L., Gourgou-Bourgade S., de la Fouchardière C. (2011). FOLFIRINOX *vs.* gemcitabine for metastatic pancreatic cancer. N. Engl. J. Med..

[38] Von Hoff D.D., Ervin T.J., Arena F.P., Chiorean G., Infante J.R., Moore M.J., Seay T.E., Tjulandin S., Ma W.W., Saleh M.N. (2012). Randomized phase III study of weekly nab-paclitaxel plus gemcitabine *vs.* gemcitabine alone in patients with metastatic adenocarcinoma of the pancreas (MPACT). J. Clin. Oncol..

[39] Ren Y.X., Xu G.M., Li Z.S., Song Y.J. (2004). Detection of point mutation in Kras oncogene at codon 12 in pancreatic diseases. World J. Gastroenterol..

[40] Cohen S.J., Ho L., Ranganathan S., Abbruzzese J.L., Alpaugh R.K., Beard M., Lewis N.L., McLaughlin S., Rogatko A., Perez-Ruixo J.J. (2003). Phase II and pharmacodynamic study of the farnesyltransferase inhibitor R115777 as initial therapy in patients with metastatic pancreatic adenocarcinoma. J. Clin. Oncol..

[41] Macdonald J.S., McCoy S., Whitehead R.P., Iqbal S., Wade J.L., Giguere J.K., Abbruzzese J.L. (2005). A phase II study of farnesyl transferase inhibitor R115777 in pancreatic cancer: A southwest oncology group (SWOG 9924) study. Investig. New Drugs.

[42] Van Cutsem E., van de Velde H., Karasek P., Oettle H., Vervenne W.L., Szawlowski A., Schoffski P., Post S., Verslype C., Neumann H. (2004). Phase III trial of gemcitabine plus tipifarnib compared with gemcitabine plus placebo in advanced pancreatic cancer. J. Clin. Oncol..

[43] Martin N.E., Brunner T.B., Kiel K.D., DeLaney T.F., Regine W.F., Mohiuddin M., Rosato E.F., Haller D.G., Stevenson J.P., Smith D. (2004). A phase I trial of the dual farnesyltransferase and geranylgeranyltransferase inhibitor L-778,123 and radiotherapy for locally advanced pancreatic cancer. Clin. Cancer Res..

[44] Gjertsen M.K., Buanes T., Rosseland A.R., Bakka A., Gladhaug I., Søreide O., Eriksen J.A., Møller M., Baksaas I., Lothe R.A. (2001). Intradermal ras peptide vaccination with granulocyte macrophage colony-stimulating factor as adjuvant: Clinical and immunological responses in patients with pancreatic adenocarcinoma. Int. J. Cancer.

[45] Muscarella P., Wilfong L.S., Ross S.B., Richards D.A., Raynov J., Fisher W.E., Flynn P.J., Whiting S.H., Rosemurgy A., Harrell F.E. (2012). A randomized, placebo controlled, double blind, multicenter phase 2 adjuvant trial of the efficacy, immunogeneicity, and safety of GI-4000 plus Gem *vs.* Gem alone in patients with resected pancreas cancer with activating Ras mutations/survival and immunology analysis of the R1 Subgroup. J. Clin. Oncol..

[46] Navas C., Hernández-Porras I., Schuhmacher A.J., Sibilia M., Guerra C., Barbacid M. (2012). Egf receptor signaling is essential for Kras oncogene-driven pancreatic ductal adenocarcinoma. Cancer Cell.

[47] Ardito C.M., Grüner B.M., Takeuchi K.K., Lubeseder-Martellato C., Teichmann N., Mazur P.K., Delgiorno K.E., Carpenter E.S., Halbrook C.J., Hall J.C. (2012). EGF receptor is required for KRAS-induced pancreatic tumorigenesis. Cancer Cell.

[48] Xiong H.Q., Rosenberg A., LoBuglio A., Schmidt W., Wolff R.A., Deutsch J., Needle M., Abbruzzese J.L. (2004). Cetuximab, a monoclonal antibody targeting the epidermal factor receptor, in combination with gemcitabine for advanced pancreatic cancer: A multicenter phase II trial. J. Clin. Oncol..

[49] Cascinu S., Berardi R., Labianca R., Siena S., Falcone A., Aitini E., Barni S., Di Costanzo F., Dapretto E., Tonini G. (2008). Cetuximab plus gemcitabine and cisplatin compared with gemcitabine and cisplatin alone in patients with advanced pancreatic cancer: A randomised, multicentre, phase II trial. Lancet Oncol..

[50] Philip P.A., Benedetti J., Corless C.L., Wong R., O’Reilly E.M., Flynn P.J., Rowland K.M., Atkins J.N., Mirtsching B.C., Rivkin S.E. (2010). Phase III study comparing gemcitabine plus cetuximab *vs.* gemcitabine in patients with advanced pancreatic adenocarcinoma: Southwest oncology group—Directed intergroup trial S0205. J. Clin. Oncol..

[51] Mohammed A., Janakiram N.B., Li Q., Madka V., Ely M., Lightfoot S., Crawford H., Steele V.E., Rao C.V. (2010). The epidermal growth factor receptor inhibitor gefitinib prevents the progression of pancreatic lesions to carcinoma in a conditional LSL-Kras^G12D^ transgenic mouse model. Cancer Prev. Res..

[52] Molina M.A., Sitja-Arnau M., Lemoine M.G., Frazier M.L., Sinicrope F.A. (1999). Increased cyclooxygenase-2 expression in human pancreatic carcinomas and cell lines: Growth inhibition by nonsteroidal anti-inflammatory drugs. Cancer Res..

[53] Tucker O.N., Dannenberg A.J., Yang E.K., Zhang F., Teng L., Daly J.M., Soslow R.A., Masferrer J.L., Woerner B.M., Koki A.T. (1999). Cyclooxygenase-2 expression is up-regulated in human pancreatic cancer. Cancer Res..

[54] Koshiba T., Hosotani R., Miyamoto Y., Wada M., Lee J.U., Fujimoto K., Tsuji S., Nakajima S., Doi R., Imamura M. (1999). Immunohisto-chemical analysis of cyclooxygenase-2 expression in pancreatic tumors. Int. J. Pancreatol..

[55] Okami J., Yamamoto H., Fujiwara Y., Tsujie M., Kondo M., Noura S., Oshima S., Nagano H., Dono K., Umeshita K. (1999). Overexpression of cyclooxygenase-2 in carcinoma of the pancreas. Clin. Cancer Res..

[56] Yip-Schneider M.T., Barnard D.S., Billings S.D., Cheng L., Heilman D.K., Lin A., Marshall S.J., Crowell P.L., Marshall M.S., Sweeney C.J. (2000). Cyclooxygenase-2 expression in human pancreatic adenocarci-nomas. Carcinogenesis.

[57] Kokawa A., Kondo H., Gotoda T., Ono H., Saito D., Nakadaira S., Kosuge T., Yoshida S. (2001). Increased expression of cyclooxygenase-2 in human pancreatic neoplasms and potential for chemoprevention by cyclooxygenase inhibitors. Cancer.

[58] Maitra A., Ashfaq R., Gunn C.R., Rahman A., Yeo C.J., Sohn T.A., Cameron J.L., Hruban R.H., Wilentz R.E. (2002). Cyclooxygenase 2 Expression in Pancreatic Adenocarcinoma and Pancreatic Intraepithelial Neoplasia: An Immunohistochemical Analysis With Automated Cellular Imaging. Am. J. Clin. Pathol..

[59] El-Rayes B.F., Zalupski M.M., Shields A.F., Ferris A.M., Vaishampayan U., Heilbrun L.K., Venkatramanamoorthy R., Adsay V., Philip P.A. (2005). A phase II study of celecoxib, gemcitabine, and cisplatin in advanced pancreatic cancer. Investig. New Drugs.

[60] Ferrari V., Valcamonico F., Amoroso V., Simoncini E., Vassalli L., Marpicati P., Rangoni G., Grisanti S., Tiberio G.A., Nodari F. (2006). Gemcitabine plus celecoxib (GECO) in advanced pancreatic cancer: A phase II trial. Cancer Chemother. Pharmacol..

[61] Morak M.J., Richel D.J., van Eijck C.H., Nuyttens J.J., van der Gaast A., Vervenne W.L., Padmos E.E., Schaake E.E., Busch O.R., van Tienhoven G. (2011). Phase II trial of Uracil/Tegafur plus leucovorin and celecoxib combined with radiotherapy in locally advanced pancreatic cancer. Radiother. Oncol..

[62] Mukherjee P., Basu G.D., Tinder T.L., Subramani D.B., Bradley J.M., Arefayene M., Skaar T., de Petris G. (2009). Progression of pancreatic adenocarcinoma is significantly impeded with a combination of vaccine and COX-2 inhibition. J. Immunol..

[63] Yip-Schneider M.T., Wu H., Hruban R.H., Lowy A.M., Crooks P.A., Schmidt C.M. (2013). Efficacy of dimethylaminoparthenolide and sulindac in combination with gemcitabine in a genetically engineered mouse model of pancreatic cancer. Pancreas.

[64] Funahashi H., Satake M., Dawson D., Huynh N.A., Reber H.A., Hines O.J., Eibl G. (2007). Delayed progression of pancreatic intraepithelial neoplasia in a conditional Kras^G12D^ mouse model by a selective cyclooxygenase-2 inhibitor. Cancer Res..

[65] Rao C.V., Mohammed A., Janakiram N.B., Li Q., Ritchie R.L., Lightfoot S., Vibhudutta A., Steele V.E. (2012). Inhibition of pancreatic intraepithelial neoplasia progression to carcinoma by nitric oxide-releasing aspirin in p48^Cre/+^-LSL-Kras^G12D/+^ mice. Neoplasia.

[66] Steinhilber D., Fischer A.S., Metzner J., Steinbrink S.D., Roos J., Ruthardt M., Maier T.J. (2010). 5-Lipoxygenase: Underappreciated role of a pro-inflammatory enzyme in tumorigenesis. Front. Pharmacol..

[67] Ding X.Z., Hennig R., Adrian T.E. (2003). Lipoxygenase and cyclooxygenase metabolism: New insights in treatment and chemoprevention of pancreatic cancer. Mol. Cancer.

[68] Funk C.D. (2001). Prostaglandins and leukotrienes: Advances in eicosanoid biology. Science.

[69] Peters-Golden M., Brock T.G. (2001). Intracellular compartmentalization of leukotriene synthesis: Unexpected nuclear secrets. FEBS Lett..

[70] Hennig R., Grippo P., Ding X.Z., Rao S.M., Buchler M.W., Friess H., Talamonti M.S., Bell R.H., Adrian T.E. (2005). 5-Lipoxygenase, a marker for early pancreatic intraepithelial neoplastic lesions. Cancer Res..

[71] Mohammed A., Janakiram N.B., Madka V., Brewer M., Ritchie R.L., Lightfoot S., Kumar G., Sadeghi M., Patlolla J.M., Yamada H.Y. (2015). Targeting pancreatitis blocks tumor-initiating stem cells and pancreatic cancer progression. Oncotarget.

[72] Brunner T.B., Geiger M., Grabenbauer G.G., Lang-Welzenbach M., Mantoni T.S., Cavallaro A., Sauer R., Hohenberger W., McKenna W.G. (2008). Phase I trial of the human immunodeficiency virus protease inhibitor nelfinavir and chemoradiation for locally advanced pancreatic cancer. J. Clin. Oncol..

[73] Ito D., Fujimoto K., Mori T., Kami K., Koizumi M., Toyoda E., Kawaguchi Y., Doi R. (2006). *In vivo* antitumor effect of the mTOR inhibitor CCI-779 and gemcitabine in xenograft models of human pancreatic cancer. Int. J. Cancer.

[74] Javle M.M., Shroff R.T., Xiong H., Varadhachary G.A., Fogelman D., Reddy S.A., Davis D., Zhang Y., Wolff R.A., Abbruzzese J.L. (2010). Inhibition of the mammalian target of rapamycin (mTOR) in advanced pancreatic cancer: Results of two phase II studies. BioMed Cent. Cancer.

[75] Mohammed A., Qian L., Janakiram N.B., Lightfoot S., Steele V.E., Rao C.V. (2012). Atorvastatin delays progression of pancreatic lesions to carcinoma by regulating PI3/AKT signaling in p48Cre/1 LSL-KrasG12D/1 mice. Int. J. Cancer.

[76] Liao J., Chung Y.T., Yang A.L., Zhang M., Li H., Zhang W., Yan L., Yang G.Y. (2013). Atorvastatin Inhibits Pancreatic Carcinogenesis and Increases Survival in LSL-KrasG12D-LSL-Trp53R172H-Pdx1-Cre Mice. Mol. Carcinog..

[77] Mohammed A., Janakiram N.B., Brewer M., Ritchie R.L., Marya A., Lightfoot S., Steele V.E., Rao C.V. (2013). Antidiabetic drug metformin prevents progression of pancreatic cancer by targeting in part cancer stem cells and mTOR signaling. Transl. Oncol..

[78] Yu Z., Zhong W., Tan Z.M., Wang L.Y., Yuan Y.H. (2015). Gemcitabine adjuvant therapy for resected pancreatic cancer: A meta-analysis. Am. J. Clin. Oncol..

[79] Reni M., Cereda S., Milella M., Novarino A., Passardi A., Mambrini A., Di Lucca G., Aprile G., Belli C., Danova M. (2013). Maintenance sunitinib or observation in metastatic pancreatic adenocarcinoma: A phase II randomised trial. Eur. J. Cancer.

[80] Sporn M.B. (1980). Combination chemoprevention of cancer. Nature.

[81] Frei E. (1972). Combination cancer therapy: Presidential address. Cancer Res..

[82] Morton J.P., Karim S.A., Graham K., Timpson P., Jamieson N., Athineos D., Doyle B., McKay C., Heung M.Y., Oien K.A. (2010). Dasatinib inhibits the development of metastases in a mouse model of pancreatic ductal adenocarcinoma. Gastroenterology.

[83] Fendrich V., Chen N.M., Neef M., Waldmann J., Buchholz M., Feldmann G., Slater E.P., Maitra A., Bartsch D.K. (2010). The angiotensin-I-converting enzyme inhibitor enalapril and aspirin delay progression of pancreatic intraepithelial neoplasia and cancer formation in a genetically engineered mouse model of pancreatic cancer. Gut.

[84] Liby K.T., Royce D.B., Risingsong R., Williams C.R., Maitra A., Hruban R.H., Sporn M.B. (2010). Synthetic triterpenoids prolong survival in a transgenic mouse model of pancreatic cancer. Cancer Prev. Res..

[85] Husain K., Centeno B.A., Chen D.T., Fulp W.J., Perez M., Zhang Lee G., Luetteke N., Hingorani S.R., Sebti S.M., Malafa M.P. (2013). Prolonged survival and delayed progression of pancreatic intraepithelial neoplasia in Pdx1^Cre^-LSL-Kras^G12D^ mice by vitamin E δ-tocotrienol. Carcinogenesis.

[86] Husain K., Centeno B.A., Chen D.T., Hingorani S.R., Sebti S.M., Malafa M.P. (2013). Vitamin E δ-Tocotrienol Prolongs Survival in the LSL-Kras^G12D^; LSL-Trp53^R172H^; Pdx-1-Cre(KPC) Transgenic Mouse Model of Pancreatic Cancer. Cancer Prev. Res..

[87] Miyabayashi K., Ijichi H., Mohri D., Tada M., Yamamoto K., Asaoka Y., Ikenoue T., Tateishi K., Nakai Y., Isayama H. (2013). Erlotinib prolongs survival in pancreatic cancer by blocking gemcitabine-induced MAPK signals. Cancer Res.

[88] Courtin A., Richards F.M., Bapiro T.E., Bramhall J.L., Neesse A., Cook N., Krippendorff B.F., Tuveson D.A., Jodrell D.I. (2013). Anti-tumour efficacy of capecitabine in a genetically engineered mouse model of pancreatic cancer. PLoS ONE.

[89] Mohammed A., Janakiram N.B., Brewer M., Biddick L., Lightfoot S., Steele V.E., Rao C.V. (2012). Targeting COX-LOX and EGFR pathways simultaneously by licofelone and gefitinib lead to complete blockade of progression of PanINs to pancreatic ductal adenocarcinoma. Cancer Res..

[90] Spano J.P., Chodkiewicz C., Maurel J., Wong R., Wasan H., Barone C., Létourneau R., Bajetta E., Pithavala Y., Bycott P. (2008). Efficacy of gemcitabine plus axitinib compared with gemcitabine alone in patients with advanced pancreatic cancer: An open-label randomized phase II study. Lancet.

[91] Kindler H.L., Ioka T., Richel D., Bennouna J., Létourneau R., Okusaka T., Funakoshi A., Furuse J., Park Y.S., Ohkawa S. (2011). Axitinib plus gemcitabine *vs.* placebo plus gemcitabine in patients with advanced pancreatic adenocarcinoma: A double-blind randomized phase 3 study. Lancet Oncol..

[92] O’Reilly E.M., Niedzwiecki D., Hall M., Hollis D., Bekaii-Saab T., Pluard T., Douglas K., Abou-Alfa G.K., Kindler H.L., Schilsky R.L. (2010). A cancer and leukemia group B phase II study of sunitinib malate in patients with previously treated metastatic pancreatic adenocarcinoma (CALGB 80603). Oncologist.

[93] Nakai Y., Isayama H., Ijichi H., Sasaki T., Sasahira N., Hirano K., Kogure H., Kawakubo K., Yagioka H., Yashima Y. (2010). Inhibition of renin-angiotensin system affects prognosis of advanced pancreatic cancer receiving gemcitabine. Br. J. Cancer.

[94] Song H., Han B., Park C.K., Kim J.H., Jeon J.Y., Kim I.G., Kim H.J., Jung J.Y., Kim J.H., Kwon J.H. (2013). Phase II trial of gemcitabine and S-1 for patients with advanced pancreatic cancer. Cancer Chemother. Pharmacol..

[95] Ueda A., Hosokawa A., Ogawa K., Yoshita H., Ando T., Kajiura S., Fujinami H., Kawai K., Nishikawa J., Tajiri K. (2013). Treatment outcome of advanced pancreatic cancer patients who are ineligible for a clinical trial. OncoTargets Ther..

[96] Heinrich S., Kraft D., Staib-Sebler E., Schwarz W., Gog C., Vogl T., Lorenz M. (2013). Phase II Study on Combined Intravenous and Intra-Arterial Chemotherapy with Gemcitabine and Mitomycin C in Patients with Advanced Pancreatic Cancer. Hepatogastroenterology.

[97] Moretto R., Raimondo L., De Stefano A., Cella C.A., Matano E., de Placido S., Carlomagno C. (2013). FOLFIRI in patients with locally advanced or metastatic pancreatic or biliary tract carcinoma: A monoinstitutional experience. Anticancer Drugs.

[98] Van Buren G., Ramanathan R.K., Krasinskas A.M., Smith R.P., Abood G.J., Bahary N., Lembersky B.C., Shuai Y., Potter D.M., Bartlett D.L. (2013). Phase II study of induction fixed-dose rate gemcitabine and bevacizumab followed by 30 Gy radiotherapy as preoperative treatment for potentially resectable pancreatic adenocarcinoma. Ann. Surg. Oncol..

[99] Chao Y., Wu C.Y., Wang J.P., Lee R.C., Lee W.P., Li C.P. (2013). A randomized controlled trial of gemcitabine plus cisplatin *vs.* gemcitabine alone in the treatment of metastatic pancreatic cancer. Cancer Chemother. Pharmacol..

[100] Vaccaro V., Bria E., Sperduti I., Gelibter A., Moscetti L., Mansueto G., Ruggeri E.M., Gamucci T., Cognetti F., Milella M. (2013). First-line erlotinib and fixed dose-rate gemcitabine for advanced pancreatic cancer. World J. Gastroenterol..

[101] Fensterer H., Schade-Brittinger C., Müller H.H., Tebbe S., Fass J., Lindig U., Settmacher U., Schmidt W.E., Märten A., Ebert M.P. (2013). Multicenter phase II trial to investigate safety and efficacy of gemcitabine combined with cetuximab as adjuvant therapy in pancreatic cancer (ATIP). Ann. Oncol..

[102] Ma W.W., Hidalgo M. (2013). The winning formulation: The development of Paclitaxel in pancreatic cancer. Clin. Cancer Res..

[103] Zhang D.S., Wang D.S., Wang Z.Q., Wang F.H., Luo H.Y., Qiu M.Z., Wang F., Li Y.H., Xu R.H. (2013). Phase I/II study of albumin-bound nab-paclitaxel plus gemcitabine administered to Chinese patients with advanced pancreatic cancer. Cancer Chemother. Pharmacol..

[104] Ko A.H., Tempero M.A., Shan Y.S., Su W.C., Lin Y.L., Dito E., Ong A., Wang Y.W., Yeh C.G., Chen L.T. (2013). A multinational phase 2 study of nanoliposomal irinotecan sucrosofate (PEP02, MM-398) for patients with gemcitabine-refractory metastatic pancreatic cancer. Br. J. Cancer.

[105] Chung J.W., Jang H.W., Chung M.J., Park J.Y., Park S.W., Chung J.B., Song S.Y., Bang S. (2013). Folfox4 as a rescue chemotherapy for gemcitabine-refractory pancreatic cancer. Hepatogastroenterology.

[106] Kim Y.J., Lee W.J., Woo S.M., Kim T.H., Han S.S., Kim B.H., Moon S.H., Kim S.S., Koh Y.H., Park S.J. (2013). Comparison of capecitabine and 5-fluorouracil in chemoradiotherapy for locally advanced pancreatic cancer. Radiat. Oncol..

[107] Herman J.M., Fan K.Y., Wild A.T., Hacker-Prietz A., Wood L.D., Blackford A.L., Ellsworth S., Zheng L., Le D.T., de Jesus-Acosta A. (2013). Phase 2 study of erlotinib combined with adjuvant chemoradiation and chemotherapy in patients with resectable pancreatic cancer. Int. J. Radiat. Oncol. Biol. Phys..

[108] Lloyd S., Chang B.W. (2013). A comparison of three treatment strategies for locally advanced and borderline resectable pancreatic cancer. J. Gastrointest. Oncol..

[109] Kim E.J., Ben-Josef E., Herman J.M., Bekaii-Saab T., Dawson L.A., Griffith K.A., Francis I.R., Greenson J.K., Simeone D.M., Lawrence T.S. (2013). A multi-institutional phase 2 study of neoadjuvant gemcitabine and oxaliplatin with radiation therapy in patients with pancreatic cancer. Cancer.

[110] Faris J.E., Blaszkowsky L.S., McDermott S., Guimaraes A.R., Szymonifka J., Huynh M.A., Ferrone C.R., Wargo J.A., Allen J.N., Dias L.E. (2013). FOLFIRINOX in locally advanced pancreatic cancer: The Massachusetts General Hospital Cancer Center experience. Oncologist.

[111] Nakai Y., Isayama H., Ijichi H., Sasaki T., Takahara N., Ito Y., Matsubara S., Uchino R., Yagioka H., Arizumi T. (2013). A multicenter phase II trial of gemcitabine and candesartan combination therapy in patients with advanced pancreatic cancer: GECA2. Investig. New Drugs.

[112] Khalil M.A., Qiao W., Carlson P., George B., Javle M., Overman M., Varadhachary G., Wolff R.A., Abbruzzese J.L., Fogelman D.R. (2013). The addition of erlotinib to gemcitabine and cisplatin does not appear to improve median survival in metastatic pancreatic cancer. Investig. New Drugs.

[113] Rougier P., Riess H., Manges R., Karasek P., Humblet Y., Barone C., Santoro A., Assadourian S., Hatteville L., Philip P.A. (2013). Randomised, placebo-controlled, double-blind, parallel-group phase III study evaluating aflibercept in patients receiving first-line treatment with gemcitabine for metastatic pancreatic cancer. Eur. J. Cancer.

[114] El-Hadaad H.A., Wahba H.A. (2013). Oxaliplatin plus 5-fluorouracil and folinic acid (OFF) in gemcitabine-pretreated advanced pancreatic cancer: A phase II study. J. Gastrointest. Cancer.

[115] Azmy A., Abdelwahab S., Yassen M. (2013). Oxaliplatin and bolus-modulated 5-fluorouracil as a second-line treatment for advanced pancreatic cancer: Can bolus regimens replace FOLFOX When considered for second line?. ISRN Oncol..

[116] Sohal D.P., Metz J.M., Sun W., Giantonio B.J., Plastaras J.P., Ginsberg G., Kochman M.L., Teitelbaum U.R., Harlacker K., Heitjan D.F. (2013). Toxicity study of gemcitabine, oxaliplatin, and bevacizumab, followed by 5-fluorouracil, oxaliplatin, bevacizumab, and radiotherapy, in patients with locally advanced pancreatic cancer. Cancer Chemother. Pharmacol..

[117] Hubner R.A., Worsnop F., Cunningham D., Chau I. (2013). Gemcitabine plus capecitabine in unselected patients with advanced pancreatic cancer. Pancreas.

[118] Heinemann V., Ebert M.P., Laubender R.P., Bevan P., Mala C., Boeck S. (2013). Phase II randomised proof-of-concept study of the urokinase inhibitor upamostat (WX-671) in combination with gemcitabine compared with gemcitabine alone in patients with non-resectable, locally advanced pancreatic cancer. Br. J. Cancer.

[119] Welsh J.L., Wagner B.A., van’t Erve T.J., Zehr P.S., Berg D.J., Halfdanarson T.R., Yee N.S., Bodeker K.L., Du J., Roberts L.J. (2013). Pharmacological ascorbate with gemcitabine for the control of metastatic and node-positive pancreatic cancer (PACMAN): Results from a phase I clinical trial. Cancer Chemother. Pharmacol..

[120] López R., Méndez C.M., Fernández M.J., Reinoso C.R., Aldana G.Q., Fernández M.S., de LA Cámara Gómez J., López M.R., Vázquez M.R., Folgar S.C. (2013). Phase II trial of erlotinib plus capecitabine as first-line treatment for metastatic pancreatic cancer (XELTA study). Anticancer Res..

[121] Infante J.R., Arkenau H.T., Bendell J.C., Rubin M.S., Waterhouse D., Jones G.T., Spigel D.R., Lane C.M., Hainsworth J.D., Burris H.A. (2013). Lenalidomide in combination with gemcitabine as first-line treatment for patients with metastatic carcinoma of the pancreas: A Sarah Cannon Research Institute phase II trial. Cancer Biol. Ther..

[122] Gunturu K.S., Yao X., Cong X., Thumar J.R., Hochster H.S., Stein S.M., Lacy J. (2013). FOLFIRINOX for locally advanced and metastatic pancreatic cancer: Single institution retrospective review of efficacy and toxicity. Med. Oncol..

[123] Tian W., Ding W., Kim S., Xu X., Pan M., Chen S. (2013). Efficacy and safety profile of combining agents against epidermal growth factor receptor or vascular endothelium growth factor receptor with gemcitabine-based chemotherapy in patients with advanced pancreatic cancer: A meta-analysis. Pancreatology.

[124] Gourgou-Bourgade S., Bascoul-Mollevi C., Desseigne F., Ychou M., Bouché O., Guimbaud R., Bécouarn Y., Adenis A., Raoul J.L., Boige V. (2013). Impact of FOLFIRINOX compared with gemcitabine on quality of life in patients with metastatic pancreatic cancer: Results from the PRODIGE 4/ACCORD 11 randomized trial. J. Clin. Oncol..

[125] Olszewski A.J., Grossbard M.L., Chung M.S., Chalasani S.B., Malamud S., Mirzoyev T., Kozuch P.S. (2013). Phase I study of oxaliplatin in combination with gemcitabine, irinotecan and 5-fluorouracil/leucovorin (G-FLIE) in patients with metastatic solid tumors including adenocarcinoma of the pancreas. J. Gastrointest. Cancer.

[126] Rose D.P., Connolly J.M. (2000). Regulation of tumor angiogenesis by dietary fatty acids and eicosanoids. Nutr. Cancer.

[127] Ge Y., Chen Z.H., Kang Z.B., Cluette-Brown J., Laposata M., Kang J.X. (2002). Effects of adenoviral gene transfer of *C. elegans* n-3 fatty acid desaturase on the lipid profile and growth of human breast cancer cells. Anticancer Res..

[128] Yang P., Chan D., Felix E., Cartwright C., Menter D.G., Madden T., Klein R.D., Fischer S.M., Newman R.A. (2004). Formation and antiproliferative effect of prostaglandin E(3) from eicosapentaenoic acid in human lung cancer cells. J. Lipid Res..

[129] Gago-Dominguez M., Yuan J.M., Sun C.L., Lee H.P., Yu M.C. (2003). Opposing effects of dietary n-3 and n-6 fatty acids on mammary carcinogenesis: The Singapore Chinese Health Study. Br. J. Cancer.

[130] Maillard V., Bougnoux P., Ferrari P., Jourdan M.L., Pinault M., Lavillonnière F., Body G., Le Floch O., Chajès V. (2002). N-3 and N-6 fatty acids in breast adipose tissue and relative risk of breast cancer in a case-control study in Tours, France. Int. J. Cancer.

[131] Xia S., Wang J.D., Kang J.X. (2005). Decreased n-6/n-3 fatty acid ratio reduces the invasive potential of human lung cancer cells by down-regulation of cell adhesion/invasion-related genes. Carcinogenesis.

[132] Granados S., Quiles J.L., Gil A., Ramírez-Tortosa M.C. (2006). Dietary lipids and cancer. Nutr. Hospitalaria.

[133] Simopoulos A.P. (2006). Evolutionary aspects of diet, the omega-6/omega-3 ratio and genetic variation: Nutritional implications for chronic diseases. Biomed. Pharmacother..

[134] Strouch M.J., Ding Y., Salabat M.R., Melstrom L.G., Adrian K., Quinn C., Pelham C., Rao S., Adrian T.E., Bentrem D.J. (2011). A High omega-3 fatty acid diet mitigates murine pancreatic precancer development. J. Surg. Res..

[135] Mohammed A., Janakiram N.B., Brewer M., Duff A., Lightfoot S., Brush R.S., Anderson R.E., Rao C.V. (2012). Endogenous n-3 Polyunsaturated Fatty Acids delay progression of Pancreatic Ductal Adenocarcinoma in Fat-1-P48^Cre/+^-LSL-Kras^G12D/+^ mice. Neoplasia.

[136] Lashinger L.M., Harrison L.M., Rasmussen A.J., Logsdon C.D., Fischer S.M., McArthur M.J., Hursting S.D. (2013). Dietary energy balance modulation of Kras- and Ink4a/Arf^+/−^-driven pancreatic cancer: The role of insulin-like growth factor-I. Cancer Prev. Res..

[137] Lanza-Jacoby S., Yan G., Radice G., LePhong C., Baliff J., Hess R. (2013). Calorie restriction delays the progression of lesions to pancreatic cancer in the LSL-KrasG12D; Pdx-1/Cre mouse model of pancreatic cancer. Exp. Biol. Med..

[138] Lashinger L.M., Malone L.M., McArthur M.J., Goldberg J.A., Daniels E.A., Pavone A., Colby J.K., Smith N.C., Perkins S.N., Fischer S.M. (2011). Genetic reduction of insulin-like growth factor-1 mimics the anticancer effects of calorie restriction on cyclooxygenase-2-driven pancreatic neoplasia. Cancer Prev. Res..

[139] Philip B., Roland C.L., Daniluk J., Liu Y., Chatterjee D., Gomez S.B., Ji B., Huang H., Wang H., Fleming J.B. (2013). A High-Fat Diet Activates Oncogenic Kras and COX2 to Induce Development of Pancreatic Ductal Adenocarcinoma in Mice. Gastroenterology.

[140] Dawson D.W., Hertzer K., Moro A., Donald G., Chang H.H., Go V.L., Pandol S.J., Lugea A., Gukovskaya A.S., Li G. (2013). High-fat, high-calorie diet promotes early pancreatic neoplasia in the conditional KrasG12D mouse model. Cancer Prev. Res..

[141] Arshad A., Chung W.Y., Steward W., Metcalfe M.S., Dennison A.R. (2013). Reduction in circulating pro-angiogenic and pro-inflammatory factors is related to improved outcomes in patients with advanced pancreatic cancer treated with gemcitabine and intravenous omega-3 fish oil. HPB.

[142] Barber M., Fearon K. (2001). Tolerance and incorporation of a high-dose eicosapentaenoic acid diester emulsion by patients with pancreatic cancer cachexia. Lipids.

[143] Anand P., Kunnumakkara A.B., Newman R.A., Aggarwal B.B. (2007). Bioavailability of curcumin: Problems and promises. Mol. Pharmacol..

[144] Kanai M., Yoshimura K., Asada M., Imaizumi A., Suzuki C., Matsumoto S., Nishimura T., Mori Y., Masui T., Kawaguchi Y. (2011). A phase I/II study of gemcitabine-based chemotherapy plus curcumin for patients with gemcitabine-resistant pancreatic cancer. Cancer Chemother. Pharmacol..

[145] Padhye S., Banerjee S., Chavan D., Pandye S., Swamy K.V., Ali S., Li J., Dou Q.P., Sarkar F.H. (2009). Fluorocurcumins as cyclooxygenase-2 inhibitor: Molecular docking, pharmacokinetics and tissue distribution in mice. Pharm. Res..

[146] Dhillon N., Aggarwal B.B., Newman R.A., Wolff R.A., Kunnumakkara A.B., Abbruzzese J.L., Ng C.S., Badmaev V., Kurzrock R. (2008). Phase II trial of curcumin in patients with advanced pancreatic cancer. Clin. Cancer Res..

[147] Kanai M., Otsuka Y., Otsuka K., Sato M., Nishimura T., Mori Y., Kawaguchi M., Hatano E., Kodama Y., Matsumoto S. (2013). A phase I study investigating the safety and pharmacokinetics of highly bioavailable curcumin (Theracurmin^®^) in cancer patients. Cancer Chemother. Pharmacol..

[148] Bai H., Li H., Zhang W., Matkowskyj K.A., Liao J., Srivastava S.K., Yang G.Y. (2011). Inhibition of chronic pancreatitis and pancreatic intraepithelial neoplasia (PanIN) by capsaicin in Pdx1^Cre^-LSL-Kras^G12D^ mice. Carcinogenesis.

[149] Shankar S., Nall D., Tang S.N., Meeker D., Passarini J., Sharma J., Srivastava R.K. (2011). Resveratrol inhibits pancreatic cancer stem cell characteristics in human and Kras^G12D^ transgenic mice by inhibiting pluripotency maintaining factors and epithelial-mesenchymal transition. PLoS ONE.

[150] Wang J., Zhang W., Sun L., Yu H., Ni Q.X., Risch H.A., Gao Y.T. (2012). Green tea drinking and risk of pancreatic cancer: A large-scale, population-based case-control study in urban Shanghai. Cancer Epidemiol..

[151] Braga M., Bissolati M., Rocchetti S., Beneduce A., Pecorelli N., di Carlo V. (2012). Oral preoperative antioxidants in pancreatic surgery: A double-blind, randomized, clinical trial. Nutrition.

[152] Plate J.M. (2012). Advances in therapeutic vaccines for pancreatic cancer. Discov. Med..

[153] Beatty G.L., Chiorean E.G., Fishman M.P., Saboury B., Teitelbaum U.R., Sun W., Huhn R.D., Song W., Li D., Sharp L.L. (2011). CD40 Agonists alter tumor stroma and show efficacy against pancreatic carcinoma in mice and humans. Science.

[154] Leach D.R., Krummel M.F., Allison J.P. (1996). Enhancement of antitumor immunity by CTLA-4 blockade. Science.

[155] Salama A.K., Hodi F.S. (2011). Cytotoxic T-lymphocyte-associated antigen-4. Clin. Cancer Res..

[156] Royal R.E., Levy C., Turner K., Mathur A., Hughes M., Kammula U.S., Sherry R.M., Topalian S.L., Yang J.C., Lowy I. (2010). Phase 2 trial of single agent ipilimumab (anti-CTLA-4) for locally advanced or metastatic pancreatic adenocarcinoma. J. Immunother..

[157] Okazaki T., Honjo T. (2006). The PD-1-PD-L pathway in immunological tolerance. Trends Immunol..

[158] Cheever M.A., Allison J.P., Ferris A.S., Finn O.J., Hastings B.M., Hecht T.T., Mellman I., Prindiville S.A., Viner J.L., Weiner L.M. (2009). The prioritization of cancer antigens: A national cancer institute pilot project for the acceleration of translational research. Clin. Cancer Res..

[159] Hollingsworth M.A., Swanson B.J. (2004). Mucins in cancer: Protection and control of the cell surface. Nat. Rev. Cancer.

[160] Ramanathan R.K., Lee K.M., McKolanis J., Hitbold E., Schraut W., Moser A.J., Warnick E., Whiteside T., Osborne J., Kim H. (2005). Phase I study of a MUC1 vaccine composed of different doses of MUC1 peptide with SB-AS2 adjuvant in resected and locally advanced pancreatic cancer. Cancer Immunol. Immunother..

[161] Yamamoto K., Ueno T., Kawaoka T., Hazama S., Fukui M., Suehiro Y., Hamanaka Y., Ikematsu Y., Imai K., Oka M. (2005). MUC1 Peptide vaccination in patients with advanced pancreas or biliary tract cancer. Anticancer Res..

[162] Soares M.M., Mehta V., Finn O.J. (2001). Three different vaccines based on the 140-amino acid MUC1 Peptide with seven tandemly repeated tumor-specific epitopes elicit distinct immune effector mechanisms in wild-type *vs.* MUC1-transgenic mice with different potential for tumor rejection. J. Immunol..

[163] Rowse G.J., Tempero R.M., VanLith M.L., Hollingsworth M.A., Gendler S.J. (1998). Tolerance and immunity to MUC1 in a human MUC1 transgenic murine model. Cancer Res..

[164] Lepisto A.J., Moser A.J., Zeh H., Lee K., Bartlett D., McKolanis J.R., Geller B.A., Schmotzer A., Potter D.P., Whiteside T. (2008). A phase I/II study of a MUC1 peptide pulsed autologous dendritic cell vaccine as adjuvant therapy in patients with resected pancreatic and biliary tumors. Cancer Ther..

[165] Beatty P.L., Narayanan S., Gariépy J., Ranganathan S., Finn O.J. (2010). Vaccine against MUC1 antigen expressed in inflammatory bowel disease and cancer lessens colonic inflammation and prevents progression to colitis associated colon cancer. Cancer Prev. Res..

[166] Plassmeier L., Knoop R., Waldmann J., Kesselring R., Buchholz M., Fichtner-Feigl S., Bartsch D.K., Fendrich V. (2013). Aspirin prolongs survival and reduces the number of Foxp3+ regulatory T cells in a genetically engineered mouse model of pancreatic cancer. Langenbecks Arch. Surg..

[167] Liu H., Yuan S.J., Chen Y.T., Xie Y.B., Cui L., Yang W.Z., Yang D.X., Tian Y.T. (2013). Preclinical evaluation of herpes simplex virus armed with granulocyte-macrophage colony-stimulating factor in pancreatic carcinoma. World J. Gastroenterol..

[168] Beatty G.L., Torigian D.A., Chiorean E.G., Saboury B., Brothers A., Alavi A., Troxel A.B., Sun W., Teitelbaum U.R., Vonderheide R.H. (2013). A Phase I Study of an agonist CD40 monoclonal antibody (CP-870,893) in combination with gemcitabine in patients with advanced pancreatic ductal adenocarcinoma. Clin. Cancer Res..

[169] Le D.T., Lutz E., Uram J.N., Sugar E.A., Onners B., Solt S., Zheng L., Diaz L.A., Donehower R.C., Jaffee E.M. (2013). Evaluation of ipilimumab in combination with allogeneic pancreatic tumor cells transfected with a GM-CSF gene in previously treated pancreatic cancer. J. Immunother..

[170] Homma Y., Taniguchi K., Nakazawa M., Matsuyama R., Mori R., Takeda K., Ichikawa Y., Tanaka K., Endo I. (2014). Changes in the immune cell population and cell proliferation in peripheral blood after gemcitabine-based chemotherapy for pancreatic cancer. Clin. Transl. Oncol..

[171] Yutani S., Komatsu N., Yoshitomi M., Matsueda S., Yonemoto K., Mine T., Noguchi M., Ishihara Y., Yamada A., Itoh K. (2013). A phase II study of a personalized peptide vaccination for chemotherapy-resistant advanced pancreatic cancer patients. Oncol. Rep..

[172] Ikemoto T., Shimada M., Iwahashi S., Saito Y., Kanamoto M., Mori H., Morine Y., Imura S., Utsunomiya T. (2014). Changes of immunological parameters with administration of Japanese Kampo medicine (Juzen-Taihoto/TJ-48) in patients with advanced pancreatic cancer. Int. J. Clin. Oncol..

[173] Hardacre J.M., Mulcahy M., Small W., Talamonti M., Obel J., Krishnamurthi S., Rocha-Lima C.S., Safran H., Lenz H.J., Chiorean E.G. (2013). Addition of algenpantucel-L immunotherapy to standard adjuvant therapy for pancreatic cancer: A phase 2 study. J. Gastrointest. Surg..

[174] Niu L., Chen J., He L., Liao M., Yuan Y., Zeng J., Li J., Zuo J., Xu K. (2013). Combination treatment with comprehensive cryoablation and immunotherapy in metastatic pancreatic cancer. Pancreas.

[175] McCaffery I., Tudor Y., Deng H., Tang R., Suzuki S., Badola S., Kindler H.L., Fuchs C.S., Loh E., Patterson S.D. (2013). Putative predictive biomarkers of survival in patients with metastatic pancreatic adenocarcinoma treated with gemcitabine and ganitumab, an IGF1R inhibitor. Clin. Cancer Res..

[176] Takahashi R., Hirata Y., Sakitani K., Nakata W., Kinoshita H., Hayakawa Y., Nakagawa H., Sakamoto K., Hikiba Y., Ijichi H. (2013). Therapeutic effect of c-Jun N-terminal kinase inhibition on pancreatic cancer. Cancer Sci..

[177] Chugh R., Sangwan V., Patil S.P., Dudeja V., Dawra R.K., Banerjee S., Schumacher R.J., Blazar B.R., Georg G.I., Vickers S.M. (2012). A preclinical evaluation of Minnelide as a therapeutic agent against pancreatic cancer. Sci. Transl. Med..

[178] Lampson B.L., Kendall S.D., Ancrile B.B., Morrison M.M., Shealy M.J., Barrientos K.S., Crowe M.S., Kashatus D.F., White R.R., Gurley S.B. (2012). Targeting eNOS in pancreatic cancer. Cancer Res..

[179] Singh M., Lima A., Molina R., Hamilton P., Clermont A.C., Devasthali V., Thompson J.D., Cheng J.H., Bou Reslan H., Ho C.C. (2010). Assessing therapeutic responses in Kras mutant cancers using genetically engineered mouse models. Nat. Biotechnol..

[180] Singh M., Couto S.S., Forrest W.F., Lima A., Cheng J.H., Molina R., Long J.E., Hamilton P., McNutt A., Kasman I. (2012). Anti-VEGF antibody therapy does not promote metastasis in genetically engineered mouse tumour models. J. Pathol..

[181] Plentz R., Park J.S., Rhim A.D., Abravanel D., Hezel A.F., Sharma S.V., Gurumurthy S., Deshpande V., Kenific C., Settleman J. (2009). Inhibition of gamma-secretase activity inhibits tumor progression in a mouse model of pancreatic ductal adenocarcinoma. Gastroenterology.

[182] Feldmann G., Maitra A. (2008). Molecular Genetics of Pancreatic Ductal Adenocarcinomas and Recent Implications for Translational Efforts. J. Mol. Diagn..

[183] Eser S., Reiff N., Messer M., Seidler B., Gottschalk K., Dobler M., Hieber M., Arbeiter A., Klein S., Kong B. (2013). Selective Requirement of PI3K/PDK1 Signaling for Kras Oncogene-Driven Pancreatic Cell Plasticity and Cancer. Cancer Cell.

[184] Mohammed A., Janakiram N.B., Madka V., Ritchie R.L., Brewer M., Biddick L., Patlolla J.M., Sadeghi M., Lightfoot S., Steele V.E. (2014). Eflornithine (DFMO) prevents progression of pancreatic cancer by modulating ornithine decarboxylase signaling. Cancer Prev. Res..

[185] Renouf D.J., Moore M.J., Hedley D., Gill S., Jonker D., Chen E., Walde D., Goel R., Southwood B., Gauthier I. (2012). A phase I/II study of the Src inhibitor saracatinib (AZD0530) in combination with gemcitabine in advanced pancreatic cancer. Investig. New Drugs.

[186] Alvarez R., Musteanu M., Garcia-Garcia E., Lopez-Casas P.P., Megias D., Guerra C., Muñoz M., Quijano Y., Cubillo A., Rodriguez-Pascual J. (2013). Stromal disrupting effects of nab-paclitaxel in pancreatic cancer. Br. J. Cancer.

[187] Neesse A., Frese K.K., Bapiro T.E., Nakagawa T., Sternlicht M.D., Seeley T.W., Pilarsky C., Jodrell D.I., Spong S.M., Tuveson D.A. (2013). CTGF antagonism with mAb FG-3019 enhances chemotherapy response without increasing drug delivery in murine ductal pancreas cancer. Proc. Natl. Acad. Sci. USA.

[188] Cabral H., Murakami M., Hojo H., Terada Y., Kano M.R., Chung U.I., Nishiyama N., Kataoka K. (2013). Targeted therapy of spontaneous murine pancreatic tumors by polymeric micelles prolongs survival and prevents peritoneal metastasis. Proc. Natl. Acad. Sci. USA.

[189] Yip-Schneider M.T., Wu H., Stantz K., Agaram N., Crooks P.A., Schmidt C.M. (2013). Dimethylaminoparthenolide and gemcitabine: A survival study using a genetically engineered mouse model of pancreatic cancer. BMC Cancer.

[190] Wu C., Fernandez S.A., Criswell T., Chidiac T.A., Guttridge D., Villalona-Calero M., Bekaii-Saab T.S. (2013). Disrupting cytokine signaling in pancreatic cancer: A phase I/II study of etanercept in combination with gemcitabine in patients with advanced disease. Pancreas.

[191] Mitsunaga S., Ikeda M., Shimizu S., Ohno I., Furuse J., Inagaki M., Higashi S., Kato H., Terao K., Ochiai A. (2013). Serum levels of IL-6 and IL-1β can predict the efficacy of gemcitabine in patients with advanced pancreatic cancer. Br. J. Cancer.

